# Hidden by the name: A new fluorescent pumpkin toadlet from the *Brachycephalus ephippium* group (Anura: Brachycephalidae)

**DOI:** 10.1371/journal.pone.0244812

**Published:** 2021-04-28

**Authors:** Ivan Nunes, Carla S. Guimarães, Pedro Henrique A. G. Moura, Mariana Pedrozo, Matheus de Toledo Moroti, Leandro M. Castro, Daniel R. Stuginski, Edelcio Muscat

**Affiliations:** 1 Laboratório de Herpetologia, Instituto de Biociências, Campus do Litoral Paulista, Universidade Estadual Paulista (UNESP), São Vicente, São Paulo, Brazil; 2 Pós-Graduação em Ecologia e Conservação, Instituto de Biociências, Universidade Federal do Mato Grosso do Sul (UFMS), Campo Grande, Mato Grosso do Sul, Brazil; 3 Projeto Dacnis, São Francisco Xavier and Ubatuba, São Paulo, Brazil; Universitat Trier, GERMANY

## Abstract

Species of *Brachycephalus* has been having taxonomical issues due its morphological similarity and genetic conservatism. Herein, we describe a new species of *Brachycephalus* from the south Mantiqueira mountain range and semidecidual forests in the municipalities of Mogi das Cruzes, Campinas and Jundiaí, state of São Paulo, Brazil, based on an integrative approach. It can be distinguished from all species of the *B*. *ephippium* species group based on morphological characters (especially osteology and head shape), advertisement call and divergence in partial mitochondrial DNA gene sequences (16S). The new species is genetically similar to *B*. *margaritatus* and morphologically similar to *B*. *ephippium*. It can be differentiated from *B*. *ephippium* by the presence of dark faded spots on skull and post-cranial plates, presence of black connective tissue connective tissue scattered over dorsal musculature, parotic plate morphology, smaller snout-vent length (adult SVL: males 13.46–15.92 mm; females 16.04–17.69 mm) and 3% genetic distance. We also present natural history data and discuss the robustness of the integrative approach, geographic distribution, genetic data, behaviour, fluorescence in ontogeny, and conservation status.

## Introduction

The genus *Brachycephalus* Fitzinger, 1826, commonly known as pumpkin toadlets [1], is composed of miniaturized frogs with cryptic and aposematic species that live in the forest leaf litter and are most active during daylight [2, 3]. The specimens are endemic to the Brazilian Atlantic Forest, eastern Brazil, and are spread along almost 1700 km, from Santa Catarina to southern Bahia states [3, 4]. Regardless of genus distribution, most *Brachycephalus* species occur in restricted lowlands or mountains areas, where some representatives seem to occupy less than 100 ha of area (sky islands; see [5]). During the last ten years, researchers have looked deeper into these frogs’ diversification and biogeography [6]. Consequently, the number of endemic *Brachycephalus* species increased significantly, especially with 15 species described for the last five years (e.g., *B*. *crispus* [7]; *B*. *albolineatus* [8]; *B*. *sulfuratus* [9]*; B*. *darkside* [10]; *B*. *actaeus* [1]). Currently, there are 36 described species for this genus, with the majority divided into three groups [2, 4, 11]: the *B*. *didactylus* group (four spp.), the *B*. *ephippium* group (12 spp.), and the *B*. *pernix* group (19 spp.). Nevertheless, recent phylogenetic study did not recover *B*. *didactylus* species group as a monophyletic group [6] suggesting that this group should not be used [12]. In addition, *B*. *atelopoide* not belong to any group and currently cannot be associated with any of the known *Brachycephalus* populations [2, 13].

The *B*. *ephippium* species group is composed by *B*. *alipioi*, *B*. *bufonoides*, *B*. *crispus*, *B*. *darkside*, *B*. *ephippium*, *B*. *garbeanus*, *B*. *guarani*, *B*. *margaritatus*, *B*. *nodoterga*, *B*. *pitanga*, *B*. *toby*, and *B*. *vertebralis* [6]. They are aposematic species that occur within the 200–1900 m altitude range (most species at altitudes greater than 500 m), from São Paulo to Minas Gerais and Espírito Santo states [4, 8]. The last species described for this group was *B*. *darkside* [10] and spotlight the requirement to verify other populations previously recognized as *B*. *ephippium* occurring along mountain ranges, especially at southern Mantiqueira mountains. Despite, Clemente-Carvalho et al. [14] reported osteological differences between *B*. *ephippium* populations from São Paulo and Rio de Janeiro states. The population from São Paulo has a wider nasal cavity and larger parotic plate than the population from Rio de Janeiro [14]. Additionally, the population from Rio de Janeiro differs from the populations from São Paulo by their paravertebral plates elongated anteriorly and posteriorly and by the presence of ridges in the posterior region of the skull. The enigmatic status of *B*. *ephippium* as a species complex was also highlighted by other recent authors, suggesting that this species should be carefully examined [3, 6, 10]. Currently, molecular evidence points to three candidate species within the *B*. *ephippium* clade, one of them for the Southern portion of Serra da Mantiqueira (sp. 5 in Condez et al. [6]).

The Mantiqueira mountain range is an orogenic system originated in the Neoproterozoic (~520 Ma.) and is the highest mountain range within the Atlantic Forest domain [15]. It is distributed for ~900 km along the borders of São Paulo, Rio de Janeiro, Espírito Santo and Minas Gerais states, in southeastern Brazil. The Mantiqueira mountain range is one of the most diverse anuran areas in the Atlantic Forest domain and is considered a hotspot of anuran endemism [16]. The species richness and the high degree of endemism in Mantiqueira mountains are partly attributed to factors such as: altitudinal gradients, heterogeneity in phytophysiognomies and the presence of humid environments along the mountain chain [17–19].

The Integrative Taxonomy approach [20] highlighted the importance of morphological features associated with different data sources, which was exemplified in several anuran groups [1, 10, 21]. The importance of combining more than one data source in *Brachycephalus* studies is related to the recent species divergence [22–24], granting the use of multiple lines of evidence as morphology [22], osteology [25, 26], bioacoustics [27, 28] and molecular identity [9] a gold standard approach for the delimitation of species of this genus.

Herein, we describe a new species of *Brachycephalus* from the southern Mantiqueira mountains based on an integrative approach, employing morphological, molecular (16S rRNA gene fragment), and bioacoustic data. We also provide an in-depth description of its natural history, based on data gathered during two years of field surveys at the Projeto Dacnis reserve and Reserva das Araucárias, both in São Francisco Xavier subdistrict, São José dos Campos Municipality, state of São Paulo, Brazil. Finally, we discuss the taxonomically informative characters and comment on the molecular data and conservation status.

## Materials and methods

### Ethics statement

The individuals examined were collected under the license SISBio #51898–1 conceded by “Instituto Chico Mendes de Conservação da Biodiversidade” (ICMBio), from the “Ministério do Meio Ambiente” (MMA), that also evaluates protocols for our collection and research. All collecting, holding and storage (scientific collection) procedures followed Brazilian animal care guidelines and were previously approved by the Universidade Estadual Paulista (UNESP) animal care committee (registration number CEUA IB/CLP #03/2020). All specimens were euthanized using anaesthetic overdose (Lidocaine 5%). The anaesthetic was applied to specimens’ dorsum and venter. All procedures were made after breathing movements ceased. The procedures followed the recommendations of AVMA [29] and CFMV [30].

### Collections and specimens examined

The specimens and audio records examined were deposited in the following Brazilian zoological collections: Fonoteca Neotropical Jacques Vielliard (FNJV), housed at Universidade Estadual de Campinas, Campinas, SP; Universidade Federal de Minas Gerais (UFMG), Amphibian Collection, Belo Horizonte, MG; Museu Nacional, Universidade Federal do Rio de Janeiro (MNRJ), Rio de Janeiro, RJ; Museu de Zoologia João Moojen de Oliveira, Universidade Federal de Viçosa (MZUFV), Viçosa, MG; Museu de Zoologia, Universidade de São Paulo (MZUSP), São Paulo, SP; Coleção Zoológica da Universidade Federal do Mato Grosso do Sul (ZUFMS-AMP), Campo Grande, MS. Almost all the acronyms and institutional abbreviations follow Sabaj [31]. The exceptions are from Coleção Científica do Laboratório de Zoologia da Universidade de Taubaté (CCLZU), Taubaté, SP; and Coleção de Anfíbios do Laboratório de Herpetologia (LHERP), Universidade Estadual Paulista (UNESP), Instituto de Biociências (HCLP-A), São Vicente, SP. All the examined specimens are listed in S1 File. All the literature used for the comparisons of morphological attributes are presented in the comparisons section.

### Morphometry

Morphological terminology of the snout shape follows Heyer et al. [32]. We used 15 measurements in the account, recorded in millimetres. Seven measurements follow Duellman [33]: Snout–vent length (SVL), head length (HL), head width (HW), eye diameter (ED), interorbital distance (IOD), internarial distance (IND) and tibia length (TL). Upper arm length (UAL), forearm length (FAL), hand length (HAL), thigh length (THL) and shank length (SHL), follows Heyer et al. [32]. Foot length (FL) was taken including the tarsus. Nostril diameter (ND) and eye–nostril distance (END) follows Napoli [34]. Snout–vent length (SVL) was taken with a calliper (precision 0.02 mm). All others were taken using the ZEISS ZEN Digital Imaging Software and a ZEISS Discovery V8 stereomicroscope (precision 0.01 mm) with an attached ZEISS Axiocam ERc 5s (5 MP) camera. ND was taken by the axis of largest diameter of the structure. Individuals HCLP-A 078, 079 and 081 could not be measured properly since they were damaged (dehydration) prior fixation. Sex of individuals was determined by jointly evaluating the presence of vocal slits and testicles in dissected males, and the presence of oocytes in dissected females, which is also externally evidenced by transparency and their overall body shape. We also took sexual size dimorphism into account. Besides their smaller size, juvenile individuals lack hyperossification or present less developed bony elements when compared with adults [10, 35].

### Bioacoustics

We recorded the calls of five males from São Francisco Xavier subdistrict, São José dos Campos Municipality, state of São Paulo, Brazil: FNJV 44407–44408/no voucher and FNJV 44409/voucher HCLP-A 260 from the type locality (Reserva das Araucárias), recorded on the 5 September 2019 at 08:30 h using Tascam DR-05 digital recorder; and from Projeto Dacnis reserve, FNJV 44406/no voucher, 8 August 2018 at 09:00 h; FNJV 44410/no voucher, 10 October 2019 at 07:30 h, both using a Motorola G7, app Audio Recorder, and FNJV 45921/voucher HCLP-A 287, 30 October 2020 at 10:00 h, using Tascam DR-05 digital recorder. All recordings with sampling rate 44.1 Hz and 16 bits resolution. We performed bioacoustic analysis using software Raven Pro 1.5 [36] with the following spectrogram settings: window type = Hann, window size = 512 samples, overlap = 80%, DFT size = 1024 samples, grid spacing = 46.9 Hz. We measured temporal and spectral parameters directly from the oscillogram and spectrogram, respectively, using measurements from Raven Pro 1.5.

Sound graphics were obtained using Seewave [37] and tuneR [38] packages from R plataform [39] and featured the following spectrogram parameters: Hann sampling window, FFT = 512, and 70% overlap. Call parameters follow the proposal of Köhler et al. [40]: note-centred approach, note duration (ND), inter-note interval (NI), note rate (NR), number of pulses per note (PN), pulse duration (PD), interpulse duration (ID), pulse rate (PR), and dominant frequency (DF). The DF was acquired with “Peak frequency” parameter from Raven Pro 1.5 [41] and represents the frequency value that corresponds to the peak of energy within the call envelope. Our results were compared with call descriptions available for the *Brachycephalus ephippium* group: *B*. *bufonoides* (see [12]), *B*. *crispus* (see [7]), *B*. *darkside* (see [10]), and *B*. *pitanga* (see [42]).

### Osteology

Osteological descriptions were based on cleared-and-double-stained specimens with Alizarin red and Alcian blue (to distinguish bone and cartilage, respectively) following the developed protocol of Taylor & Van Dyke [43]. We prepared three specimens (ZUFMS-AMP 13647–13649; one juvenile and two male adults, respectively) under this procedure. Terminology of the bone elements follows Silva et al. [44] and Campos et al. [35]. We removed the skin of the specimens after the result of cleared-and-double-stained (and there were no osteoderms on the skin).

### Genetic distances

We extracted whole genomic DNA from ethanol-preserved muscle tissue according to the following protocol: approximately 1 mm of tissue sample was put in a sterile Eppendorf and were added 50 μL of EAR buffer (100 mM of TrisHCl pH 8.5; 5 mM of EDTA pH 8.0; 0.2% of SDS and 200 mM of NaCl) and 4 μL of proteinase K (20 mg/mL). The sample was incubated overnight at 55°C, under 300 rpm shaking. Then, the sample was incubated for 10 minutes at 95°C without agitation, followed by the addition of 500 μL of TE buffer (1 mM of EDTA; 10 mM of TrisHCl, pH 7.5). Finally, 5 μL of RNAse A (3mg/mL in TE buffer) was added and incubation was done for 10 minutes at 37°C. The final sample was stored at -20°C. Primers (An16SF 5’–ACCGTGCGAAGGTAGCGTAATC–3’; and An16SR 5’–CCTGATCCAACATCGAGGTCG–3’) and DNA amplification (PCR) procedures followed Lyra et al. [45]. Sequencing was performed at BPI Biotecnologia Ltda. (Botucatu, state of São Paulo, Brazil). A fragment of the 16S rRNA gene was sequenced for three specimens of the new species (HCLP-A 078–080, GenBank accession numbers: MT396578, MT396579 and MT396580, respectively) resulting in 416 bp.

We edited and aligned with sequences of seven species from *Brachycephalus ephippium* species group available in GenBank in Geneious v.7.1.3 with the MUSCLE algorithm using default parameters [46]. We removed the gaps using Gblocks [47, 48]. The final dataset was 410 base pairs (bp; 98% of the original 416 positions). We have calculated sequence divergence (uncorrected p-distances) among species/individuals for the 16S mtDNA using MEGA v.7.0.26 [49]. To assess the sequence divergence of the new species within the genus *Brachycephalus ephippium* species group, homologous sequences of remaining species of this group available in GenBank were downloaded (S1 Table).

### Natural history

We studied the natural history of the new species from October 2017 to September 2019 in two different areas in São Francisco Xavier subdistrict, São José dos Campos, state of São Paulo, Brazil (Projeto Dacnis Reserve and Reserva das Araucárias). São Francisco Xavier is located in the Mantiqueira Mountains with typical montane and submontane Atlantic Forest formations (750 to 2000 m a.s.l.) with patches of rocky outcrops and many rocky streams. The area has a seasonality with the dry season occurring from April to August (average temperature in July is 15.5°C) and the wet season between September and March (average temperature in January is 21.6°C) (http://climate-data.org). In 2018, the pluviometric index ranged from 26 mm in June to 444 mm in November (private pluviometer located near Projeto Dacnis area; brand Acurite 01079 Pro Weather Station). To statistically analyse the influence of rainfall on the abundance of sighted individuals of *Brachycephalus* **sp. nov.** monthly, we used a generalized linear model (GLM) with a Poisson regression in software R [39]. This test is suitable for parametric data and count data.

During the study we conducted 76 active search field surveys between October 2018 and September 2019, totalling 94 hours/man. We also used additional data from fortuitous observations made from October 2017 to September 2018. Our search effort was made during daylight hours, mostly between 08:00 and 11:00 am. We collected data on seasonal and daily activity, microhabitat use, breeding activity, and defensive behaviours of the specimens. For the purpose of discussing conservation issues, we have calculated the Extent of Occurrence (EOO; [50]) using the tool “Polygon” on Google Earth Pro^©^ (Google LLC). EOO is calculated by applying a *Minimum Convex Polygon* (MCP), the smallest polygon in which no internal angle exceeds 180 degrees, and which contains all the sites of occurrence [50].

### Registration of nomenclatural act

The electronic version of this article in Portable Document Format (PDF) will represent a published work according to the International Commission on Zoological Nomenclature (ICZN), and hence the new names contained in the electronic version are effectively published under that Code from the electronic edition alone. This published work and the nomenclatural acts it contains have been registered in ZooBank, the online registration system for the ICZN. The ZooBank Life Science Identifiers (LSIDs) can be resolved and the associated information viewed through any standard web browser by appending the LSID to the prefix http://zoobank.org/. The LSID for this publication is: urn:lsid:zoobank.org:pub:08CB9413-3F67-4B23-8D71-214B604B46FD. The online version of this work is archived and available from the following digital repositories: PLOS One, PubMed Central, and CLOCKSS.

## Results

### Description of the new species

#### *Brachycephalus rotenbergae* sp. Nov

**Fig 1:**
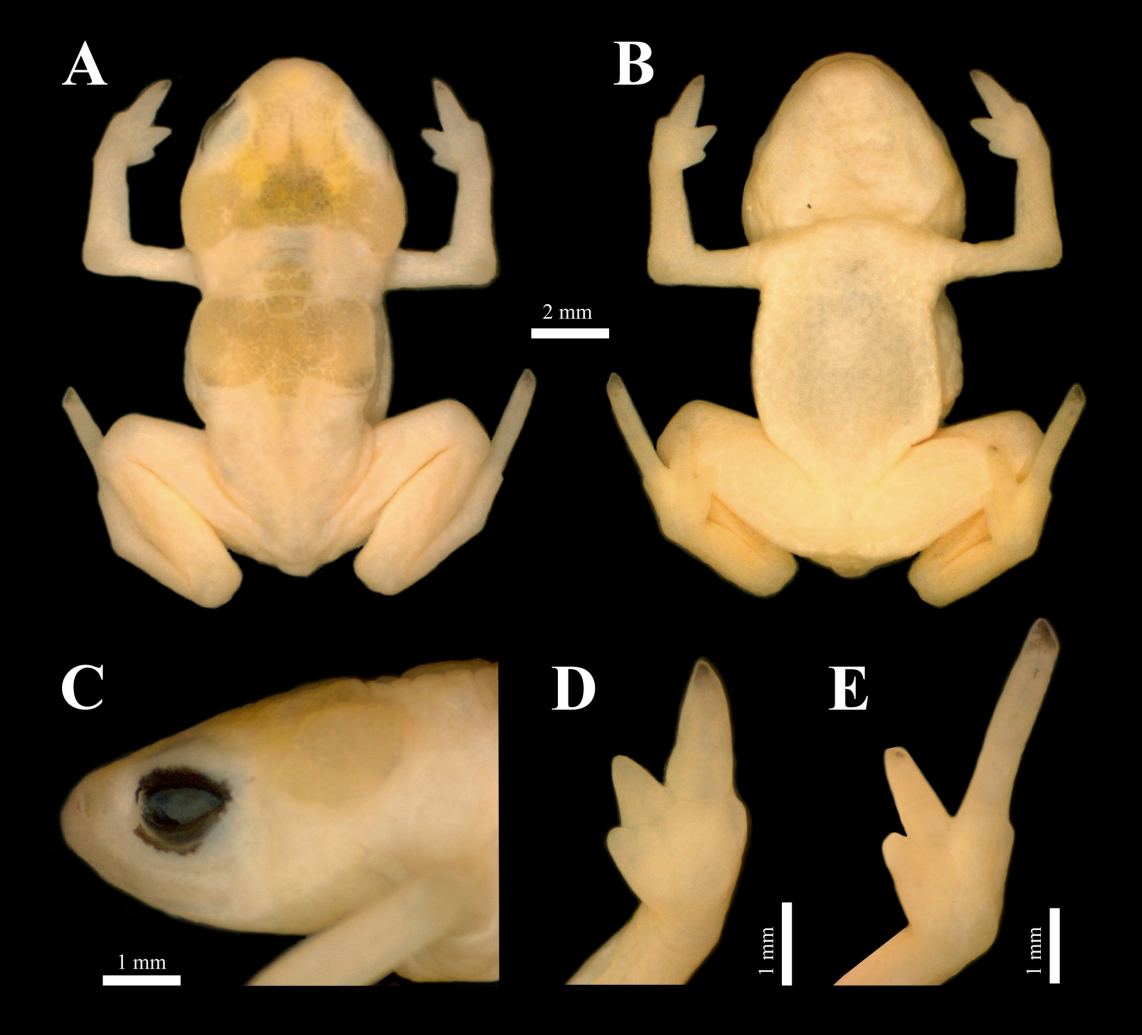
*Brachycephalus rotenbergae* sp. nov., holotype (HCLP-A 260, adult male, SVL = 13.5 mm). (A) Dorsal view, (B) ventral view, (C) lateral view of head, (D) ventral view of hand, (E) ventral view of feet.

**Fig 2:**
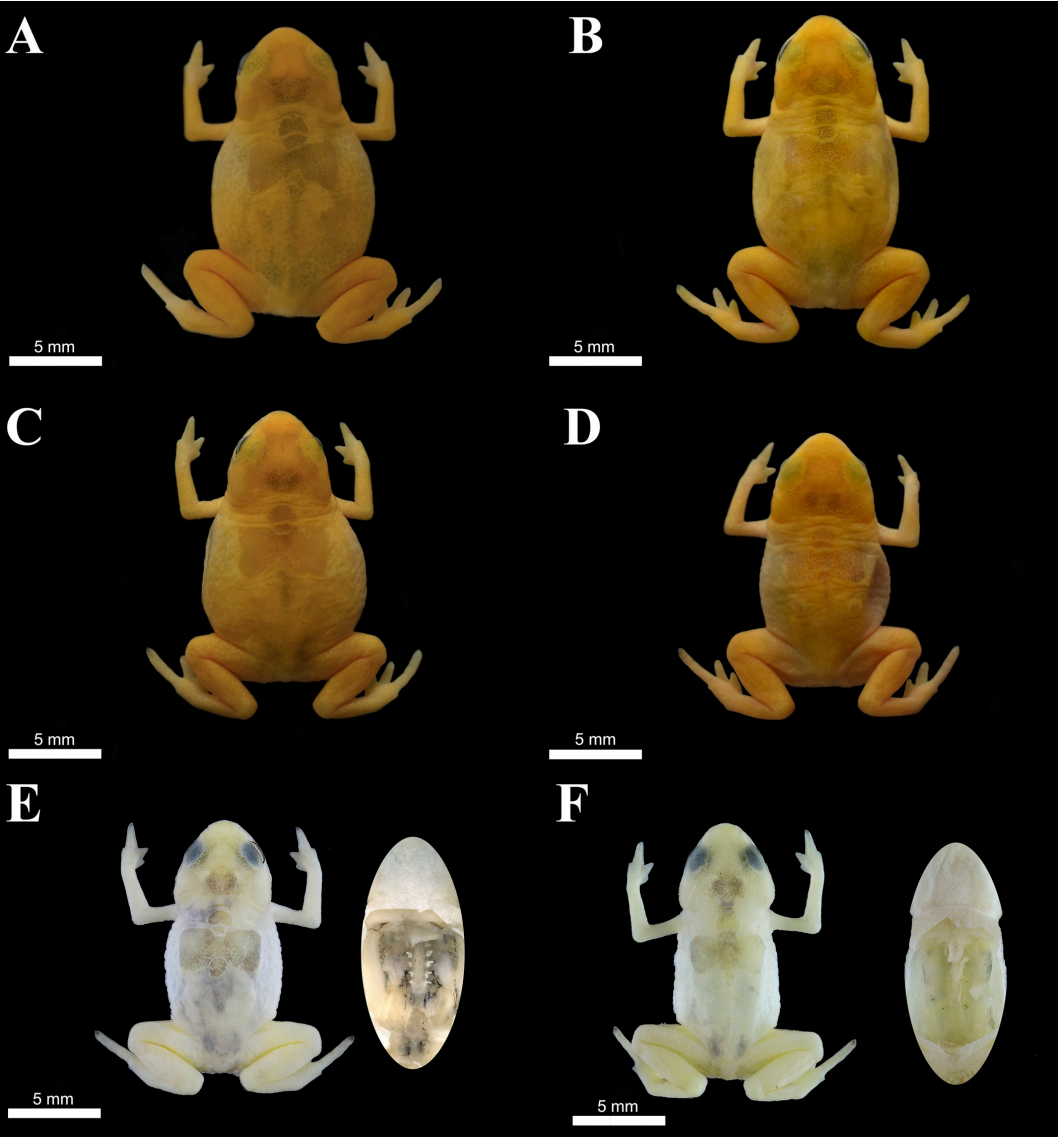
Variation in specimens of *Brachycephalus rotenbergae* **sp. nov.** regarding dorsal shield shape (A-D) and amount of black connective tissue (E-F). (A) HCLP-A 284 (adult female), (B) HCLP-A 285 (adult female), (C) HCLP-A 286 (adult female), (D) HCLP-A 287 (adult male), (E) ZUFMS-AMP 13648 (adult male), (F) ZUFMS-AMP 13649 (adult male).

(Figs 1 and 2) urn:lsid:zoobank.org:act:4BC1043A-A0CD-4CF9-AE1B-3B242A4B42AA

*Brachycephalus ephippium* (populations from São Francisco Xavier subdistrict, São José dos Campos, state of São Paulo, Brazil) [14]

*Brachycephalus ephippium* [51]

*Brachycephalus ephippium* [52]

*Brachycephalus ephippium* [53]

*Brachycephalus* sp. 5 [6]

#### Holotype

HCLP-A 260 (Fig 1), adult male, SVL 13.5 mm, collected at “Reserva das Araucárias” (22^o^ 54’ 11.3" S, 46^o^ 00’ 59" W; 1929m a.s.l.; Datum WGS-84), São Francisco Xavier subdistrict, São José dos Campos Municipality, state of São Paulo, Brazil, on 05 September 2019 by E. Muscat, D.R. Stuginski, M.T. Moroti, and M. Pedrozo.

#### Paratypes

HCLP-A 078–081 (adult specimens) collected on 15 March 2019 by E. Muscat. HCLP-A 082–083 (adult male and female respectively) collected, on 20 February 2019 by E. Muscat, D.R. Stuginski, P.H.A.G Moura and I. Nunes. HCLP-A 287, adult male, collected on 30 October 2020 by E. Muscat and M.T. Moroti. All collected at Projeto Dacnis reserve (22° 53’ 44" S, 45° 56’ 08" W, 840m a.s.l.; Datum WGS-84), São Francisco Xavier subdistrict, São José dos Campos Municipality, São Paulo state, Brazil. HCLP-A 284–286, adult females collected at type locality, on 30 October 2020 by E. Muscat and M.T. Moroti. CCLZU 1274, 1278 (adult females) and 1279 (adult male) collected at RPPN “O Primata” (former “Fazenda Mandala”), São Francisco Xavier subdistrict, São José dos Campos Municipality, state of São Paulo, Brazil (22° 53’ 44"S, 45° 58’ 04"W, 1000m a.s.l.; Datum WGS-84), on 04 May 2002 by T. Bagatim. ZUFMS-AMP 13647–13649 (one juvenile and two adult specimens, respectively) cleared and double-stained, collected at type locality, on 8 July 2019 by E. Muscat.

#### Non-type material

HCLP-A 281–283 (adult specimens), no sex determined due to dehydration, collected at Projeto Dacnis reserve (22° 53’ 44" S, 45° 56’ 08" W), São Francisco Xavier subdistrict, São José dos Campos Municipality, state of São Paulo, Brazil, on 20 March 2019 by E. Muscat.

### Diagnosis

*Brachycephalus rotenbergae* **sp. nov.** can be assigned to the genus *Brachycephalus* by observed phalangeal reduction, an arciferal pectoral girdle in which the ossified procoracoid and epicoracoid cartilages are fused to the clavicle, coracoid, and scapula, a suprascapula expanded with a prominent cleithrum, and the absence of a sternum (putative synapomorphies; see Hedges et al. [54]). A member of the *B*. *ephippium* species group by the presence of dermal co-ossification (putative synapomorphy; see [55]). The new species can be diagnosed from its congeners by the following combination of characters: (1) bufoniform body; (2) bright orange overall body coloration; (3) presence of skull and post-cranial plates; (4) presence of faded dark spots over skull and post-cranial plates; (5) absence of metacarpal and metatarsal tubercles; (6) rounded snout; (7) paravertebral plates forming a trapezoidal bony shield; (8) absence of osteoderms; (9) laterally expanded parotic plates; (10) flat paravertebral plates; (11) paravertebral plates not projected over vertebral spines; (12) presence of black connective tissue scattered over dorsal musculature; (13) head wider than long (mean HL/HW ~ 90% for both male and females); (14) adult SVL 13.46–15.92 mm for males and 16.04–17.69 mm for females; (15) advertisement call with dominant frequency of 2.84–4.52 kHz, note duration of 0.13–014 s, and at rate 2.50–3.42 note/s, each note had 8–13 pulses, emitted at rate 50.56–71.82 pulses/s.

### Comparisons with other species

**Fig 3:**
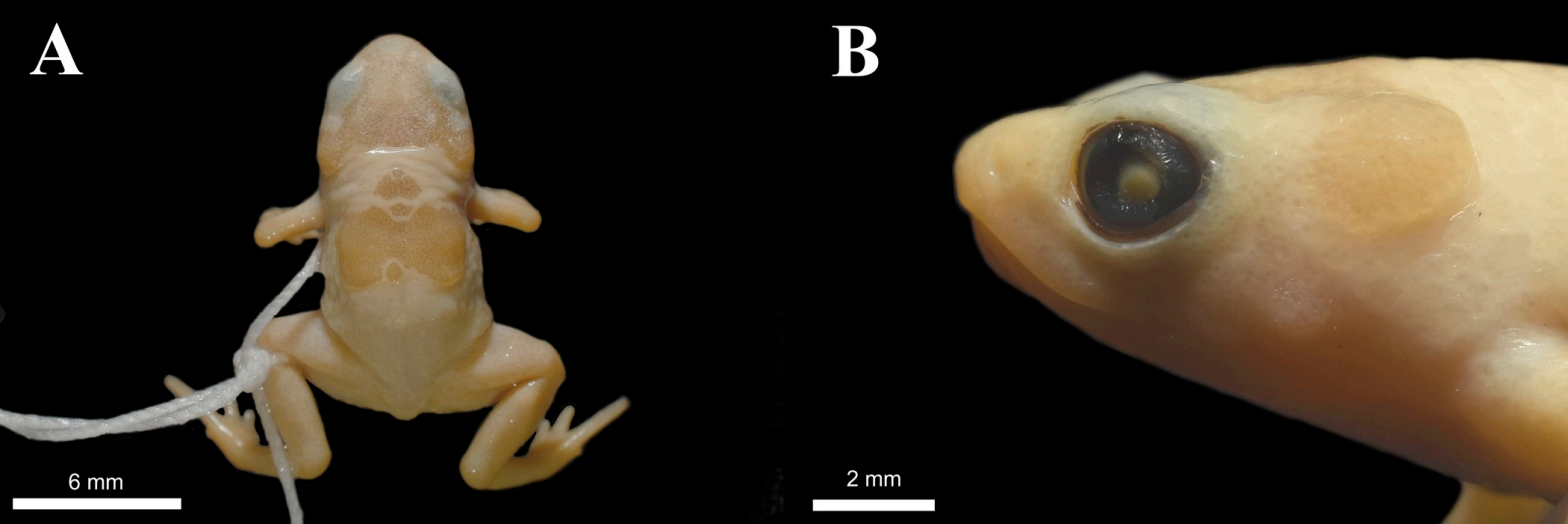
*Brachycephalus ephippium* from the species type locality (MNRJ 40806, adult male, SVL 16.26 mm). (A) Dorsal view, and (B) lateral view of head.

The bufoniform body of *Brachycephalus rotenbergae* **sp. nov.** distinguishes it from *Brachycephalus didactylus*, *B*. *hermogenesi*, *B*. *pulex*, and *B*. *sulfuratus*, which have leptodactyliform bodies [9, 26, 56, 57]. The bright orange overall body coloration of *B*. *rotenbergae* **sp. nov.** differentiates from species that present coloured stripes, spots or blotches (as in *B*. *actaeus*, *B*. *auroguttatus*, *B*. *boticario*, *B*. *coloratus*, *B*. *crispus*, *B*. *ferruginus*, *B*. *fuscolineatus*, *B*. *guarani*, *B*. *hermogenesi*, *B*. *izecksohni*, *B*. *leopardus*, *B*. *mariaterezae*, *B*. *mirissimus*, *B*. *pernix*, *B*. *pitanga* and *B*. *pombali*, [1, 2, 5, 7, 11, 56–61]), brownish coloration (*B*. *brunneus*, *B*. *curupira*, *B*. *didactylus*, *B*. *nodoterga*, *B*. *pulex*, *B*. *quiririensis*, *B*. *sulfuratus* [9, 11, 26, 55, 56, 58, 62], and green background in (*B*. *albolineatus*, *B*. *olivaceus*, *B*. *toby*, *B*. *verrucosus*, *B*. *tridactylus* [2, 8, 27, 28]). In preservative, *Brachycephalus rotenbergae* **sp. nov.** presents a yellowish-cream coloration with faded dark spots over cranial and post-cranial plates and dorsal dark background. This distinguishes it from other species, with coloration in preservative cream in *B*. *alipioi*, *B*. *bufonoides* and *B*. *ephippium* (Fig 3); and greyish-cream in *B*. *garbeanus* and *B*. *margaritatus*, [12, 13, 55, 63, 64]. The dark background in *B*. *darkside* is more pronounced [10]. The externally distinct, although reduced, finger IV differentiates the new species from *B*. *tridactylus* (finger IV not externally distinct in this species [28]).

*Brachycephalus rotenbergae* **sp. nov.** also exhibits black connective tissue scattered over dorsal musculature, which in most specimens also forms two dorsally visible paired marks on the sacral region (Fig 2E and 2F). This feature distinguishes this species from *B*. *ephippium*, *B*. *izecksohni*, *B*. *pernix*, and *B*. *pitanga*, all lacking this pigmentation. In *B*. *garbeanus* and *B*. *margaritatus*, some specimens were reported to present few scattered areas with pigmentation in the dorsolateral region adjacent to the dorsal musculature. While in *B*. *vertebralis* there is some internal dark pigmentation near the region of the spinal vertebrae [10].

The presence of hyperossification and skin with dermal ossification on the head and dorsum distinguishes the new species from the *B*. *pernix* species group (*B*. *actaeus*, *B*. *albolineatus*, *B*. *auroguttatus*, *B*. *boticario*, *B*. *brunneus*, *B*. *coloratus*, *B*. *curupira*, *B*. *ferruginus*, *B*. *fuscolineatus*, *B*. *izecksohni*, *B*. *leopardus*, *B*. *mariaterezae*, *B*. *mirissimus*, *B*. *olivaceus*, *B*. *pernix*, *B*. *pombali*, *B*. *quiririensis*, *B*. *tridactylus* and *B*. *verrucosus*, [1, 2, 5, 8, 11, 28, 58, 60, 62, 65]), and from *B*. *didactylus*, *B*. *hermogenesi*, *B*. *pulex* and *B*. *sulfuratus*, all of which lacking hyperossification [9, 26, 56, 57]. The parotic plates of *B*. *rotenbergae* **sp. nov.** are expanded laterally, making the posterior region of head wider than long, and partially concealing the posterior tip of the squamosal. In *B*. *ephippium*, the parotic plates are not expanded laterally, making the head longer than wide, while they are more developed anteriorly (CSG pers. obs.; also see [10, 35]).

The presence of paravertebral plates forming a trapezoidal bony shield in *Brachycephalus rotenbergae* **sp. nov.** differentiates it from *B*. *alipioi*, *B*. *atelopoide*, *B*. *bufonoides*, *B*. *crispus*, *B*. *guarani*, *B*. *nodoterga*, *B*. *pitanga*, *B*. *toby* and *B*. *vertebralis* (absent in these species [7, 12, 13, 27, 55, 59, 61, 63, 66]). The absence of osteoderms distinguishes *B*. *rotenbergae* **sp. nov.** from *B*. *crispus*, *B*. *margaritatus* and *B*. *nodoterga* [7, 55, 64]. A flat dorsal shield distinguishes the new species from *B*. *darkside*, *B*. *garbeanus* and *B*. *margaritatus*, which have convex shields [10, 13, 55, 64]. Moreover, the paravertebral plates of *B*. *rotenbergae* **sp. nov.**, although fused with the spinal plates forming a shield, is not projected over the vertebral spine. This characteristic further distinguishes it from *B*. *garbeanus*, that exhibits paravertebral plates that are projected over the vertebral spine [64]. The absence of warts on body also differentiates the new species from *B*. *atelopoide* [13, 55].

Snout shape rounded in dorsal and lateral views in *Brachycephalus rotenbergae* **sp. nov.** differentiates it from *B*. *didactylus*, *B*. *hermogenesi* and *B*. *pulex* (snout pointed in dorsal view [26, 56, 57]), *B*. *brunneus* (snout slightly mucronate in dorsal view [2]), *B*. *leopardus* (snout slightly truncate in dorsal and lateral views [2]), and also *B*. *quiririensis* (snout mucronate in dorsal view [62]). The protuberant nostrils of the new species also distinguishes it from *B*. *alipioi*, *B*. *auroguttatus*, *B*. *bufonoides*, *B*. *fuscolineatus*, *B*. *garbeanus*, *B*. *pernix* and *B*. *vertebralis*, all with non-protuberant nostrils [2, 12, 55, 63, 65, 66].

The absence of metacarpal and metatarsal tubercles distinguishes *B*. *rotenbergae* **sp. nov.** from *B*. *hermogenesi* (metacarpal and metatarsal tubercles present [57]) and *B*. *auroguttatus*, *B*. *boticario*, *B*. *brunneus*, *B*. *fuscolineatus*, *B*. *hermogenesi*, *B*. *izecksohni*, *B*. *leopardus*, *B*. *mariaeterezae*, *B*. *olivaceus*, *B*. *pitanga*, *B*. *pulex*, *B*. *quiririensis*, *B*. *toby* and *B*. *verrucosus* (outer metatarsal tubercles present [2, 27, 56–59, 62]). The head wider than long further distinguishes the new species from *B*. *ephippium* and *B*. *garbeanus* (head longer than wide [13]).

Among species of the *Brachycephalus ephippium* group, *B*. *rotenbergae* **sp. nov.** (male SVL 13.46–15.92 mm, female SVL 16.04–17.69 mm) is larger than *B*. *alipioi* (adult SVL 12.5–16.2 mm, [63]), *B*. *bufonoides* (male SVL 12.0–14.5 mm, female SVL 11.9–15.6 mm, [12]), *B*. *crispus* (male SVL 11.5–12.9 mm, female SVL 11.9–15.6 mm, [7]), *B*. *guarani* (adult SVL 8.7–13.4 mm, [61]), *B*. *nodoterga* (holotype SVL 12.4 mm, [13]), *B*. *pitanga* (male SVL 10.8–12.1 mm, female SVL 12.6–14.0 mm, [59]), *B*. *toby* (male SVL 11.3–12.1 mm, female SVL 13.4–14.2 mm, [27]), and *B*. *vertebralis* (adult SVL 10.5–15.1 mm, [66]). Furthermore, we note that the new species is smaller than *B*. *ephippium* individuals from its type locality (male SVL 16.26 mm, female SVL 17.23–18.99 mm, this study).

The advertisement call of *Brachycephalus rotenbergae* **sp. nov.** distinguishes it from *B*. *bufonoides* by the higher note rate (NR = 2.50–3.42 notes/s in new species; 1.98–2.43 notes/s in *B*. *bufonoides*). The advertisement call of *Brachycephalus rotenbergae* **sp. nov.** distinguishes it from *B*. *crispus* by the shorter note duration (ND = 0.09–0.24 s in new species; 0.26–0.28 s in *B*. *crispus*), shorter interval between notes (0.11–0.25 s in new species; 0.35±0.02 s in *B*. *crispus*), higher note rate (NR = 2.5–3.48 notes/s in new species; 1.67±0.09 notes/s in *B*. *crispus*) and higher pulse rate (PR = 50.56–71.82 pulses/s in new species; 17.4±2.12 pulses/s in *B*. *crispus*). It is distinguished from *B*. *darkside*, with a slight overlap, by having more pulses per note (PN = 8–13 pulses/note, 10.14±0.9, in new species; 5–8 pulses/note, 6.3±0.7, in *B*. *darkside*). See all call parameters of *Brachycephalus ephippium* species group in Table 1.

**Table 1:** Described advertisement calls of species belonging to the *Brachycephalus ephippium* group.

Call parameters	Call series duration (s)	Note duration (s)	Interval between notes (s)	Note rate (notes/s)	Number of pulses per note	Pulse rate (pulses/s)	Dominant frequency (kHz)	Locality	References
***Brachycephalus* sp. nov.**	> 93.01	0.13–0.24 (0.16 ± 0.02)	0.11–0.25 (0.15 ± 0.03)	2.50–3.48 (3.07 ± 0.46)	8–13 (9.66 ± 0.84)	50.56–71.82 (60.09 ± 3.71)	3.04–4.52 (3.42 ± 0.44)	São Francisco Xavier subdistrict, São José dos Campos Municipality, SP	This work
	120–360	0.09–0.12 (0.11 ± 0.006)	0.12–0.14 (0.13 ±0.006)	–	5–15 (12 ± 1.96)	–	3.4–5.3	Serra das Cabras, Campinas Municipality, SP	[51] (as *B*. *ephippium)*
	**> 93.01**	**0.09–0.24**	**0.11–0.25**	**2.50–3.48**	**5–15**	**50.56–71.82**	**3.04–5.3**	**Combined**	
***B*. *bufonoides***	> 180	0.22–0.30 (0.27 ± 0.018)	0.15–0.26 (0.21 ± 0.0246)	1.98–2.43 (2.15 ± 0.20)	13–17 (15.05 ± 0.88)	47.55–71.28 (56.2 ± 4.81)	4.13–4.88 (4.55 ± 0.14)	Área de Proteção Ambiental de Macaé de Cima, Nova Friburgo Municipality, RJ	[12]
***B*. *crispus***	> 300	0.26–0.28* (0.28 ± 0.02)	0.35 ± 0.02	1.67 ± 0.09	7–12 (10 ± 1.19)	17.4 ± 2.12	3.8–5.7* (4.6 ± 0.19)	Parque Estadual da Serra do Mar, Núcleo Cunha, Cunha Municipality, SP	[7]
***B*. *darkside***	2.9–66.2 (30.4 ± 25.3)	0.08–0.16 (0.11 ± 0.01)	0.12–0.21 (0.15 ± 0.01)	3.10–4.05 (3.52 ± 0.42)	5–8 (6.3 ± 0.7)	36.8–78.4 (56.9 ± 4.9)	2.8–3.8 (3.4 ± 0.2)	Parque Estadual da Serra do Brigadeiro, Ervália Municipality, MG	[10]
***B*. *pitanga***	–	0.15–0.17* (0.17 ± 0.01)	0.18–0.20*	2.65 ± 0.18	11–12* (11.1 ± 1.2)	62 ± 8	4.2–6.5* (4.9 ± 0.2)	Parque Estadual da Serra do Mar, São Luís do Paraitinga Municipality, SP	[42]

Parameters presented as range (mean ± SD). The parameter values of call references have been converted from the original data to the same unit when necessary.

* Values extracted from figures of the wave form. **

### Description of the holotype

Body robust and bufoniform; head wider than long, 42.3% (HL/SVL) and 43.2% (HW/SVL) (Fig 1A); snout short, round in dorsal and lateral views (Fig 1A and 1C); nostrils slit-shaped, protuberant and directed anterolaterally; *canthus rostralis* indistinct; loreal region slightly concave; lips curved, nearly sigmoid; eyes slightly protruding and directed anterolaterally, 27.4% (ED/HW) and 27.9% (ED/HL); tympanum indistinct; vocal sac not externally expanded; vocal slits present; tongue longer than wide, with posterior half not adhered to mouth floor; choanae small and rounded; vomerine teeth absent.

Arm and forearm relatively slender; arm slightly shorter than forearm (UAL 22.6% and FAL 23.1% of SVL); forearm slightly hypertrophied; hand shorter than forearm. Hands with all fingers distinct; fingers I and IV reduced, vestigial; II and III robust; tips of fingers I and IV rounded, II and III pointed; relative finger length IV < I < II < III; subarticular tubercles absent, inner and outer metacarpal tubercles absent (Fig 1D). Leg relatively short and moderately robust; shank slightly shorter than thigh (SHL/SVL = 38.0% and THL/SVL = 44.3%); foot longer than tarsus and shorter than shank, with toes I and V reduced, vestigial; toes II, III and IV distinct and with pointed tips; relative toe length I < V < II < III < IV; subarticular, inner and outer metatarsal tubercles absent (Fig 1E).

Skin on top of head and dorsum rough due to surface ornamentation of skull and post-cranial plates; dorsal posterior region of body with scattered warts; skin on belly and limbs smooth; skin on lateral surface of the body and around cloacal opening granular.

### Measurements of holotype (in mm)

SVL 13.46; HL 5.70; HW 5.81; ED 1.59; IOD 2.92; END 0.95; ND 0.27; IND 1.87; UAL 3.04; FAL 3.11; HAL 2.48; THL 5.95; SHL 5.11; FL 7.51.

### Colour in life

**Fig 4:**
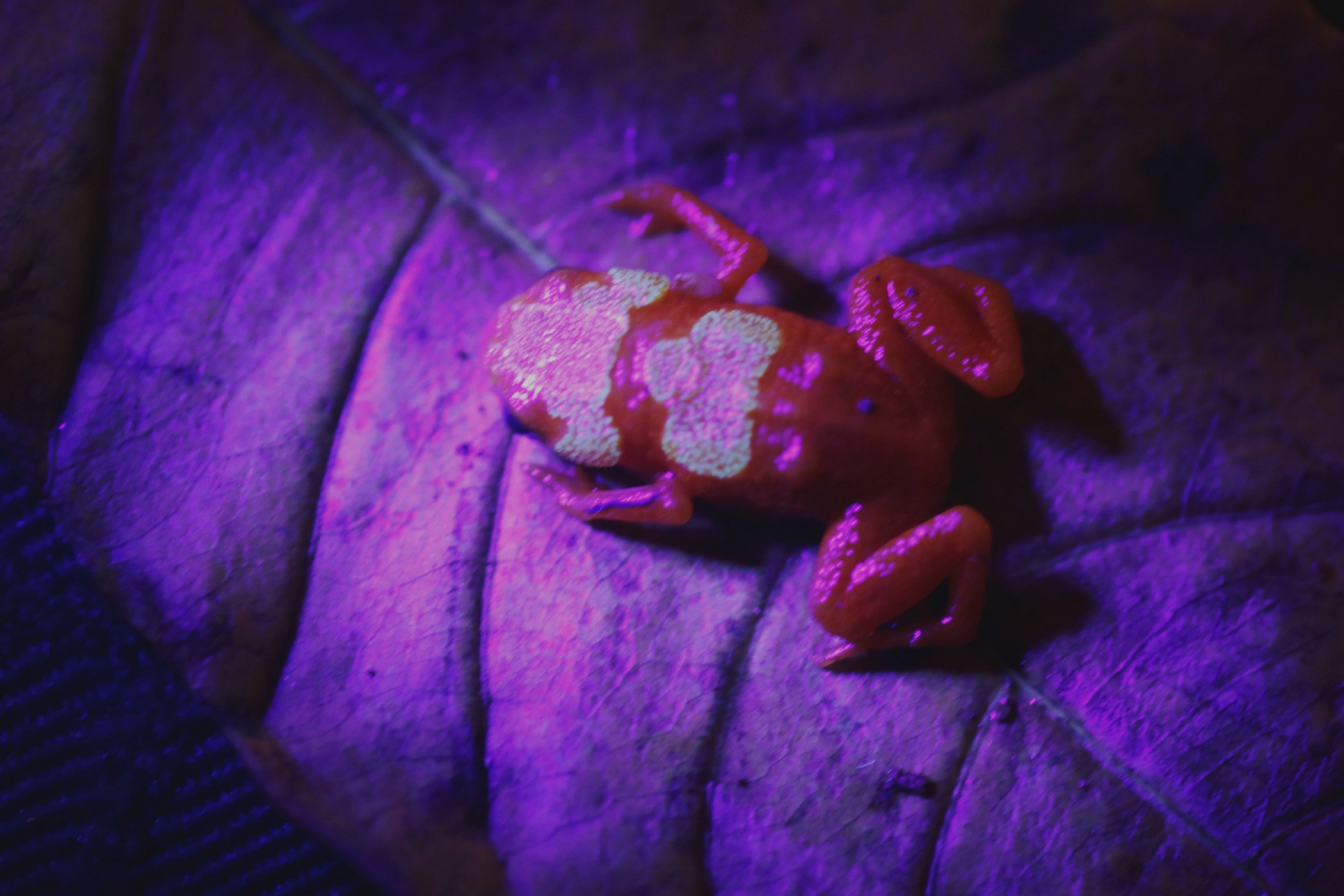
*Brachycephalus rotenbergae* sp. nov. lightened by a UV flashlight (395 nm wavelength). Deatil of fluorescence in dorsal and cephalic shields.

Overall colour of the body is bright orange. Skull and post-cranial plates darker than the background of the dorsum, with faded dark spots or shades. Eyes completely black. Like other *Brachycephalus* species, *Brachycephalus rotenbergae* **sp. nov.** presented fluorescence when backlighted by a UV flashlight (Ultrafire®, 395 nm wavelength) (Fig 4).

### Colour in preservative

Overall colour of the body is pale, yellowish cream. Belly with dark background. Tips of FIV and TIV with dark spots. Skull and dorsal plates greyish yellow with faded dark spots. Eyes completely black.

### Variation

Measurements of the type series are given in Table 2. The overall texture of the skin varies among individuals, with some specimens presenting an entirely smooth body, while others exhibit scattered warts or granular texture on the lateral portions of the trunk. Ontogenetic variation in co-ossification of skull and post-cranial plates is present, with juveniles exhibiting less ossification and ornamentation. The fluorescence at the skull and post-cranial plates cannot be seen by the naked eye in juvenile specimens (SVL = 7.0 mm). The relative size of paravertebral plates may vary between individuals with the same body size (Fig 2A–2D). Spinal plates of presacral vertebrae I and II may be separated from each other, although always in close contact. The spinal plate of presacral III may be fused to the dorsal shield. The spinal plate of presacral VIII and sacral vertebrae may be separated from the dorsal shield. The amount of black connective tissue covering the dorsal musculature may vary from just a few scattered spots (Fig 2F) to a more intense coverage (Fig 2E). Dark spots may be present or absent below the tip of FIV or TIV. Colour in life is the same among adults, but different between adults and juveniles: the juvenile specimens are slightly darker, as reported for other *Brachycephalus* species.

**Table 2:** Measurements in millimetres of the 11 adults of the type series of *Brachycephalus rotenbergae* sp. nov. *X*, mean; SD, standard deviation.

	Males (n = 5)			Females (n = 6)		
Character	*X*	SD	Range	*X*	SD	Range
**SVL**	13.68	1.04	13.46–15.92	17.06	0.71	16.04–17.69
**HL**	5.36	0.16	5.25–5.7	5.61	0.37	5.1–6.2
**HW**	5.81	0.17	5.67–6.15	5.99	0.37	5.49–6.47
**ED**	1.36	0.59	1.25–2.78	1.59	0.16	1.36–1.76
**IOD**	2.85	1.05	1.27–4.57	3.70	0.90	2.85–4.68
**END**	1.09	0.21	0.75–1.32	1.12	0.22	0.82–1.36
**ND**	0.34	0.09	0.27–0.51	0.45	0.06	0.31–0.49
**IND**	1.89	0.29	1.65–2.5	2.05	0.11	1.83–2.12
**UAL**	3.01	0.18	2.88–3.36	3.43	0.34	2.88–3.67
**FAL**	3.27	0.11	3.11–3.41	3.44	0.21	3.16–3.73
**HAL**	2.49	0.25	2.29–3.02	2.91	0.25	2.49–3.26
**THL**	5.95	0.26	5.52–6.26	6.34	0.63	5.34–7.29
**SHL**	5.22	0.30	5.06–5.85	5.64	0.54	5.03–6.4
**FL**	8.54	0.94	7.40–10.55	9.57	0.77	7.67–10.73

### Etymology

The epithet *rotenbergae* is a patronym in honor to Elsie Laura K. Rotenberg, founder, and leader of the Projeto Dacnis, a Brazilian NGO dedicated to research and conservation of Atlantic Forest domain.

### Advertisement call

**Fig 5:**
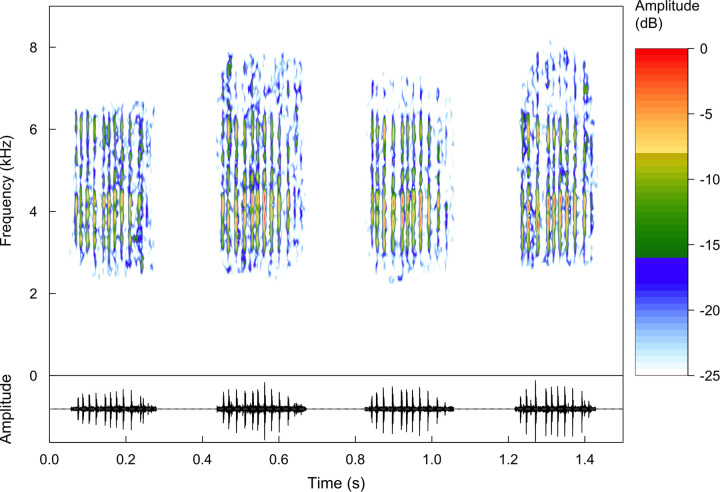
Advertisement call of the holotype of *Brachycephalus rotenbergae* sp. nov. (HCLP-A 260). Recorded on 5 September 2019 at 08:30 h (FNJV 44409). Spectrogram (above) and oscillogram (bellow).

Our recordings featured a single type of call from *Brachycephalus rotenbergae* **sp. nov.**, which is considered herein as the species advertisement call (*sensu*[67]). This call is characterized by pulsed notes (note = call) without frequency modulation and emitted in long sequences (i.e. call series, *sensu* [67]; n = 5 call series, 408 notes; Fig 5; Table 1). Our longest recording featured more than 265 notes in a series but did not encompass the beginning of such series. Note duration is 0.13–0.24 s (ND = 0.17±0.02, n = 204 notes), emitted at intervals of 0.11–0.22 s (NI = 0.18±0.02, n = 198 intervals), and at rate of 2.50–3.42 notes/s (NR = 2.98±0.39). Each note had an average of 10 pulses (PN = 8–13 pulses, 10.14±0.97, n = 204 notes, 2009 pulses) emitted at constant rate (PR = 50.56–71.82 pulses/s, 57.91±4.07). Dominant frequency ranged from 2.84–4.52 kHz (DF = 3.83±0.35).

### Osteology

**Fig 6:**
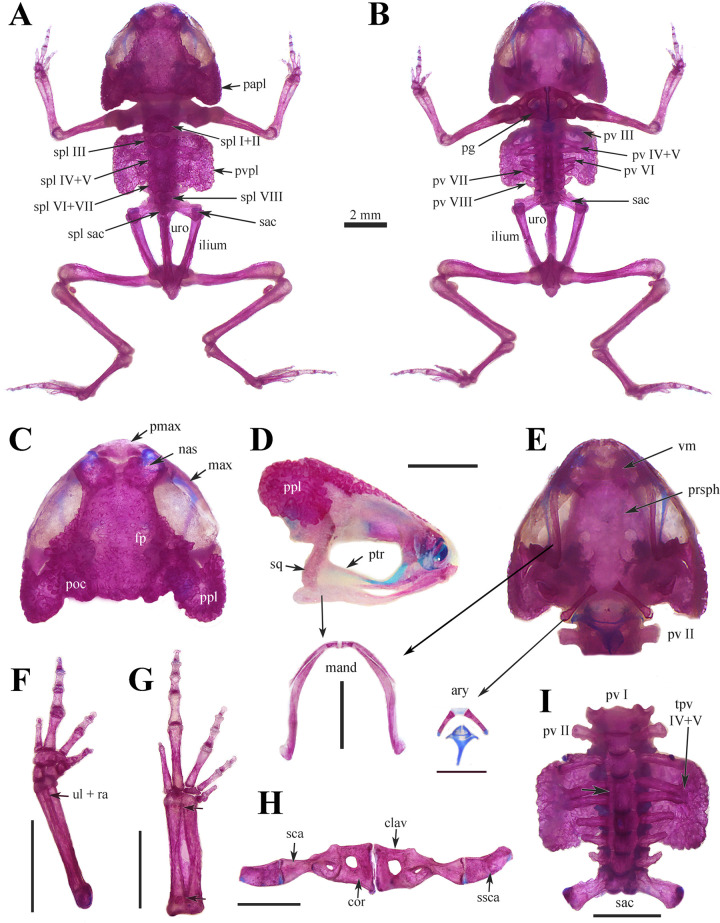
Cleared and double-stained specimens of *Brachycephalus rotenbergae* sp. nov. (A) Dorsal and (B) ventral view of complete skeleton; (C) dorsal, (D) lateral and (E) ventral view of skull with details of mandible and arytenoids cartilage; (F) dorsal view of left hand, arrow indicates ulna and radio fused; (G) dorsal view of left foot, arrow indicates tibiale and fibulare fused; (H) ventral view of pectoral girdle with scapula and supra-scapula deflected; (I) ventral view of spine, arrows indicates vertebrae IV and V fused (in vertebrae body and in transverse process). Vouchers, figs. A, B and E (ZUFMS-AMP 13648, male, SVL 15.89 mm); C, D, F, G, H and I (ZUFMS-AMP 13649, male, SVL 15.79 mm). Acronyms: ac (arytenoids cartilage), clav (clavicle), cor (coracoid), mand (mandible), max (maxilla), nas (nasal), papl (parotic plate), pg (pectoral girdle), pmax (premaxilla), poc (pos orbital crest), prsph (parasphenoid), ptr (pterigoid), pv (presacral vertebrae), pvpl (paravertebral plate), ra (radius), sac (sacrum), sca (scapula), spl (spinal plate), sq (squamosal), ssca (supra-scapula), tpv (transverse process of presacral vertebrae), ul (ulna), uro (urostyle), vm (vomer). Scale bar = 2 mm.

The two cleared and double stained specimens of *Brachycephalus rotenbergae* **sp. nov.** showed a well-developed skull and post-cranial plates, both laterally expanded. Parotic, spinal and paravertebral plates ornamented with dermal roofing bones (Fig 6A). The specimens ZUFMS-AMP 13648–13649 (males) have, respectively SVL 15.9, and 15.8 mm. Body skin of these specimens without osteoderms.

Dorsomedially, the parotic plate is fused with the post-orbital crest, and it is expanded laterally, making the posterior region of head wide; this plate partially conceals the posterior edge of squamosal and optic capsule, laterally (Fig 6A). Nasal, sphenethmoid, frontoparietals, prootics, and exoccipital fused, forming a dorsal cranial plate. All frontoparietals (periorbital, parietal and central regions) ornamented with dermal roofing bones; anterior and parietal regions of frontoparietals fused medially; lateral and posterior orbital flaps present and conspicuous; post-orbital crest present and ornamented with dermal roofing bones (Fig 6C). Premaxillae broad, not fused medially; *pars dentalis* present, but odontoids absent. Posterior end of the maxilla tapered; posterior end of angulosplenial with squamosal is projected anteriorly than the posterior line of the skull. Quadratojugal and palatine absents. Pterygoid slender, dorsoventrally oriented, anterior ramus long articulating with maxillary arch; posterior ramus short, articulating with ventral ramus of squamosal (Fig 6D). Squamosal broad in lateral view; zygomatic extremely short, and optic ramus short, both not ornamented. Nasal capsule and sphenethmoid (nasal region) completely ossified. Nasal bones hyperossified with ornamentation; anteromedial region of nasals concave and posteromedial fused. Parasphenoid robust and fused with sphenethmoid. Vomer distinct with rounded medial edges (6E). Tympanic annulus absent. Mandible edentate. Arytenoid cartilages not mineralized (Fig 6E).

Ventral column composed of eight non-imbricate vertebrae and hyperossified spinal processes of vertebrae (Fig 6B and 6I). Only the first presacral vertebra, atlas, lacks transverse process. Presence of spinal and paravertebral plates well developed and ornamented (Fig 6A). Spinal plates (spl) associated with the spinal process of all presacral (I-VIII) and sacral vertebrae. The spl I and II fused, forming broad plate as kidney shaped; spl III small with irregular shaped, separated of principal group of dorsal plates; spl IV and V fused; spl VI-VIII slightly separated each other’s with irregular shaped, and size decreasing towards the sacral region; spl of sacral vertebral fin as dagger (Fig 6A). Paravertebral plates broad with rounded edges. Dorsally, the paravertebral plates are medially fused with spinal plates IV and V (Fig 6A); anteriorly conceals (completely or partially) the transverse process of the vertebra III; medially conceals completely the transverse process of vertebra IV and V, which are fused with the paravertebral plates ventrally (Fig 6I); and posteriorly conceals (completely or partially) the transverse process of the vertebra VII (Fig 6B and 6I).

Pectoral girdle arciferal and robust (Fig 6B and 6H). Omosternum discrete (cartilaginous); sternum absent. Procaracoid and epicoracoid ossified and fused, both united with clavicle, coracoid and scapula (all fused). Clavicle and scapula fused; clavicle acromial process short; pit rounded between clavicle and coracoid. Suprascapula expanded (Fig 6H). Forearm (radius and ulna fused and distinguishable) slightly shorter than humerus (Fig 6F). Manus with distal carpals (I-V) fused, with central, radiale and ulnare about the same size. Phalangeal formula 1-2-3-1; tips of the terminal phalangeal elements of fingers III and IV rounded arrow shaped (Fig 6F). Femur and tibiofibula (tibia and fibula fused and distinguishable) of approximately the same length. Tibiale and fibulare fused at their distal and proximal ends, not fused medially (Fig 6G). Tarsal elements I, II and III absente; central present; one very reduced prehallical element. Phalangeal formula 1-2-3-4-1; tips of the terminal phalangeal elements of toes II, III and IV arrow shaped, I and V rounded (Fig 6G).

### Genetic distances

The genetic distances varied from 1–7% of divergence and depict *B*. *rotenbergae* **sp. nov**. as most similar to *B*. *margaritatus* (1–2%). *Brachycephalus rotenbergae* **sp. nov**. differs from *B*. *ephippium* by 3%; *B*. *bufonoides* by 4%; *B*. *garbeanus* by 5%; *B*. *nodoterga*, *B*. *toby*, *B*. *vertebralis* by 6–7%; *B*. *alipioi*, *B*. *crispus*, *B*. *guarani*, and *B*. *pitanga* by 7%. See S2 Table for genetic distances between species of the *B*. *ephippium* species group.

### Geographic distribution

**Fig 7:**
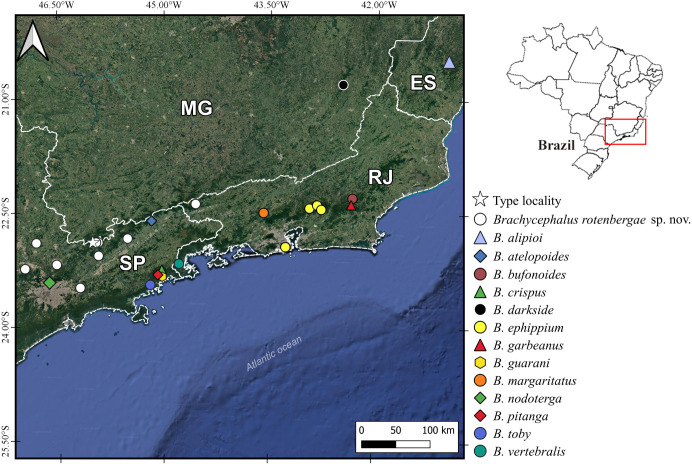
Geographic distribution map for the species of *Brachycephalus ephippium* group on Google satellite map. See the species records on S3 Table. Star = type locality of *B*. *rotenbergae* **sp. nov.** ES = state of Espírito Santo, MG = state of Minas Gerais, RJ = state of Rio de Janeiro, SP = state of São Paulo.

Currently, a monophyletic clade of four populations named *Brachycephalus* sp. 5. in the cities of Campinas, Mogi das Cruzes, Atibaia and Jundiaí, all in the state of São Paulo; and two populations from the municipality of Resende, state of Rio de Janeiro [6]. Based on molecular distance, *B*. sp. 5 is synonymous with *B*. *rotenbergae* **sp. nov**., known for 10 locations in the southern portion of Serra da Mantiqueira (Fig 7). The known distribution of *B*. *rotenbergae* **sp. nov.** is shown on the map, as well as the other type localities for species of the *B*. *ephippium* group. In the case of *B*. *ephippium*, we indicate the type locality [56, 63, 66] and the locations pointed out by Condez et al. [6].

### Natural history

#### Microhabitat structure

The field surveys were conducted in two different areas within the subdistrict. These areas present some differences in the slope of the terrain, vegetation structure, and altitude.

*Projeto Dacnis* (22°53’S 45°56’W, 842m a.s.l.). This area is composed of two land margins separated by a small stream that is at most 1.5m wide and runs slowly through the landscape. This stream is shallow, its bottom is composed mainly of sand and small rocks, and there are many logs, tree branches and plants that can be used as bridges by small animals. On one margin there is a swamp area where most of the soil is permanently wet. The canopy cover of the swamp is mostly composed of banana trees (*Musa* sp.), and light incidence is very reduced. It is surrounded by a sloping dry area with medium-high canopy cover and bushes. On this side of the stream, most *Brachycephalus rotenbergae* **sp. nov.** specimens were using the slopes, perched on bushes, twigs or roots. However, we also found other specimens moving across the muddy area of the swamp, using fallen and non-fallen banana trees as perching sites.

**Fig 8:**
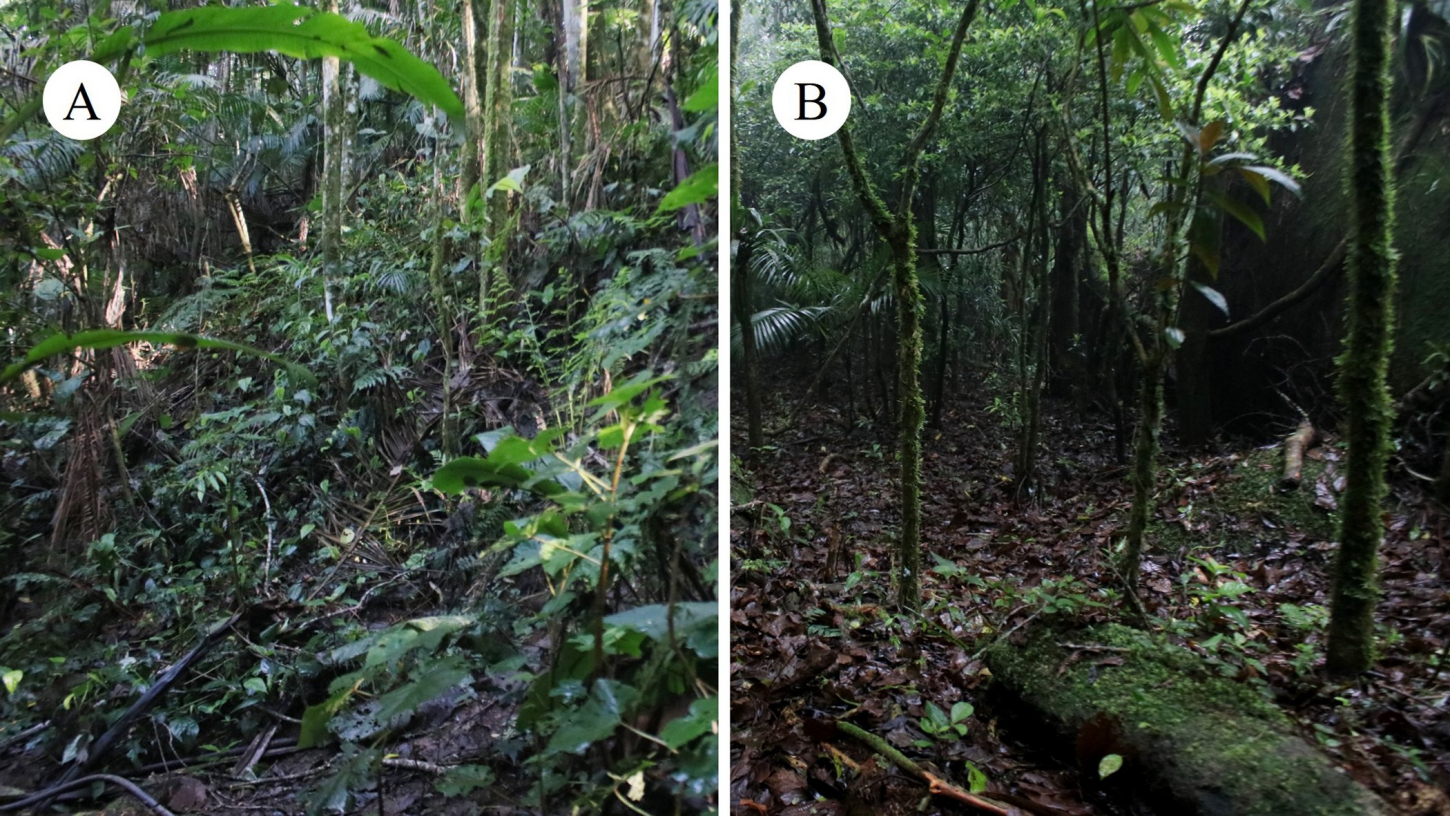
Study areas for the natural history observations of *Brachycephalus rotenbergae* sp. nov. (A) São Francisco Xavier, Projeto Dacnis, and (B) Reserva das Araucárias.

On the opposite margin of the stream there is a heavily sloped terrain with medium-high canopy cover formed by juçara palms (*Euterpe edulis*), bushes and some sparse trees (Fig 8A). The light incidence is medium–high. There is a shallow leaf litter cover with many branches and roots emerging from the soil. These are used by the specimens as perch sites during their activity, especially the dry leaves of juçara palms. Most of the specimens in this area were found perched at small heights, although others were found under leaf litter. specimens also used the root formations of juçara palms as shelter, and we also observed a reproductive activity inside these structures (see below). The exposure of the specimens on the perches seems to be linked to their seasonal activity and to the weather conditions. In dry conditions, most specimens were found under the leaf litter instead of perched.

Our observations also suggest that there is a local movement of the specimens on the slope that is linked to abiotic factors, since during dry periods we found most specimens on the lower parts of the slope (closer to the stream). However, when the wet season begins, and the soil gets wet, activity seems to occur all along the slope. The sloped terrain, the large number of perches, the presence of leaf litter and the proximity to the stream seem to influence the specimens concentration in this spot when compared to other sites in the vicinity where specimens are present only in small numbers.

*Reserva das Araucárias* (22°54.1130’S 46°0.0590’ W, 1299 m a.s.l.). This is a relatively flat, forested area with some pronounced rocky outcrops. The canopy is dense and high, and light incidence on the soil is medium–low. In the understory there are countless lianas, small bushes and many fallen twigs and logs (Fig 8B). A deep leaf litter layer lies on a muddy soil and most of the trees have abundant moss. Small streams surround the area but do not cut into it as in the Projeto Dacnis area. In Reserva das Araucárias we found more *Brachycephalus rotenbergae* **sp. nov.** specimens per day than in Projeto Dacnis area. specimens were found both perched and moving across the leaf litter. specimens used rock outcrops, fallen tree trunks, roots and twigs as perching sites. Even though the streams are not so close in Reserva das Araucárias, the environment is also very humid during the wet season. In fact, this area is usually covered in dense fog at night due to the high altitude.

In both areas, specimens were found using perches and leaf litter. Most perches were no more than 30cm high, although a male was 140 cm from the ground. Males and females share perching habits, and it seems to be more related to weather conditions than to vocalization activity. During all field surveys, we noticed that perched specimens detected our presence very quickly. We could see them trying to escape or hide under the leaf litter. Thus, even without any rigorous scientific test, we believe that *Brachycephalus rotenbergae* **sp. nov.** has good eyesight and maybe this could be linked to the perching behaviour.

#### Activity and spatial use

**Fig 9:**
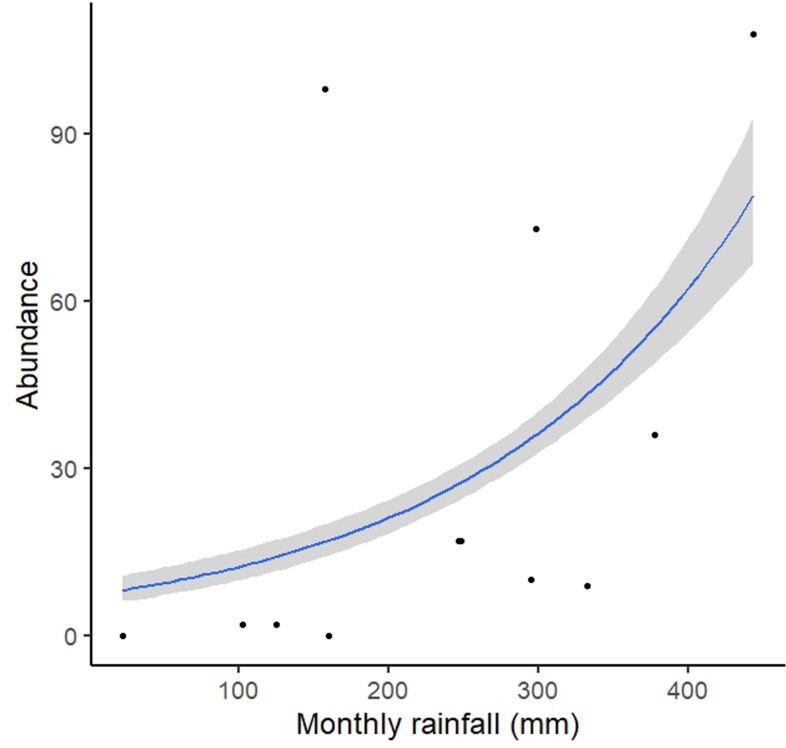
Relationship between observed *Brachycephalus rotenbergae* sp. nov. specimens and monthly precipitation (in mm). Notice that the number of specimens found per field survey reaches zero during months with precipitation under 200 mm.

The monthly abundance of individuals is positively correlated with monthly rainfall (R^2^ = 0.25; p = 0.005; Fig 9). During the study we performed 370 observations. In both areas, we were able to detect specimens almost all along the way, but in much higher numbers in specific points. *Brachycephalus rotenbergae* **sp. nov.** activity is seasonal and related to rain regime (Fig 9). specimens were found all year long, except from May to July (Table 3). The activity peak seems to occur from September to February. During the active season, specimens were found in most field surveys, but activity peaks seem to be related both to the season and to rainy weather. Thus, in the same month specimens were present in high or low numbers according to the weather conditions. In September 2019, for instance, we were able to find 38 specimens during a single field survey that occurred after almost a week of weak rain, but during successive dry days such activity decreased abruptly, and no specimens were found in this same area. Months with less than 200 mm of precipitation did not seem to be favourable to the activity of *Brachycephalus rotenbergae* **sp. nov.**, however, it is important to notice that months with low precipitation but with constant drizzling rains can present a huge specimen’s activity (Fig 9). During these months (especially at the beginning of the wet season), most specimens were found after short rain periods, when air and soil humidity increased for a little while. Although soil humidity was not measured during our study, specimens’ activity seemed to be linked to it. During extended rain periods, soil was moist, and activity seemed to occur more continuously, but during sparse rainy periods, soil moisture did not last long, and only brief peaks of activity were observed. Temperature may also affect activity, yet probably secondary to humidity, as during our field surveys we found active specimens at temperatures below 16°C coupled with drizzling rain, but no activity in warm but dry weeks.

**Table 3:** Number of observations, calling activity and amplexus during one year of field survey for the study of Natural History of *Brachycephalus rotenbergae* sp. nov.

	oct/18	nov/18	dec/18	jan/19	feb/19	mar/19	apr/19	may/19	jun/19	jul/19	aug/19	sep/19
**Number of frogs**	36	108	17	17	73	9	10	0	0	0	2	98
**Frogs/person/hour**	3.9	10.1	6.8	3.4	15.8	3.6	1.5	0.0	0.0	0.0	0.1	10.0
**Minimum frogs**	0	0	8	0	1	2	1	0	0	0	0	0
**Maximum frogs**	7	28	9	7	50	4	7	0	0	0	1	45
**Calling activity**	No	**Yes**	No	**Yes**	**Yes**	No	No	No	No	No	**Yes**	**Yes**
**Amplexus record**	No	**Yes**	No	No	**Yes**	No	No	No	No	No	No	**Yes**

The species is diurnal and only seven of 370 observations were made at night. These seven specimens were inactive and hidden under leaf litter, and no calls were heard. In our study we found *Brachycephalus rotenbergae* **sp. nov.** specimens during all daytime length, including late afternoon. We did not measure the activity of the specimens in different daylight hours, but we recorded calling activity and amplexus both in the morning and in the afternoon.

In both areas, specimens were found using perches and leaf litter. Most perches were no more than 30 cm high, although a male was 140 cm from the ground. Males and females share perching habits, and it seems to be more related to weather conditions than to vocalization activity. During all field surveys, we noticed that perched specimens detected our presence very quickly. We could see them trying to escape or hide under the leaf litter. Thus, even without any rigorous scientific test, we believe that *Brachycephalus rotenbergae* **sp. nov.** has good eyesight and maybe this could be linked to the perching behaviour.

#### Breeding activity

Calling activity seems to be seasonal and related to weather, it was recorded less than 15 times during all field surveys (Table 3). Males were found calling mostly under the leaf litter, but we also saw males perched with their legs extended and heads pointing up, in calling activity (S1 Video).

**Fig 10:**
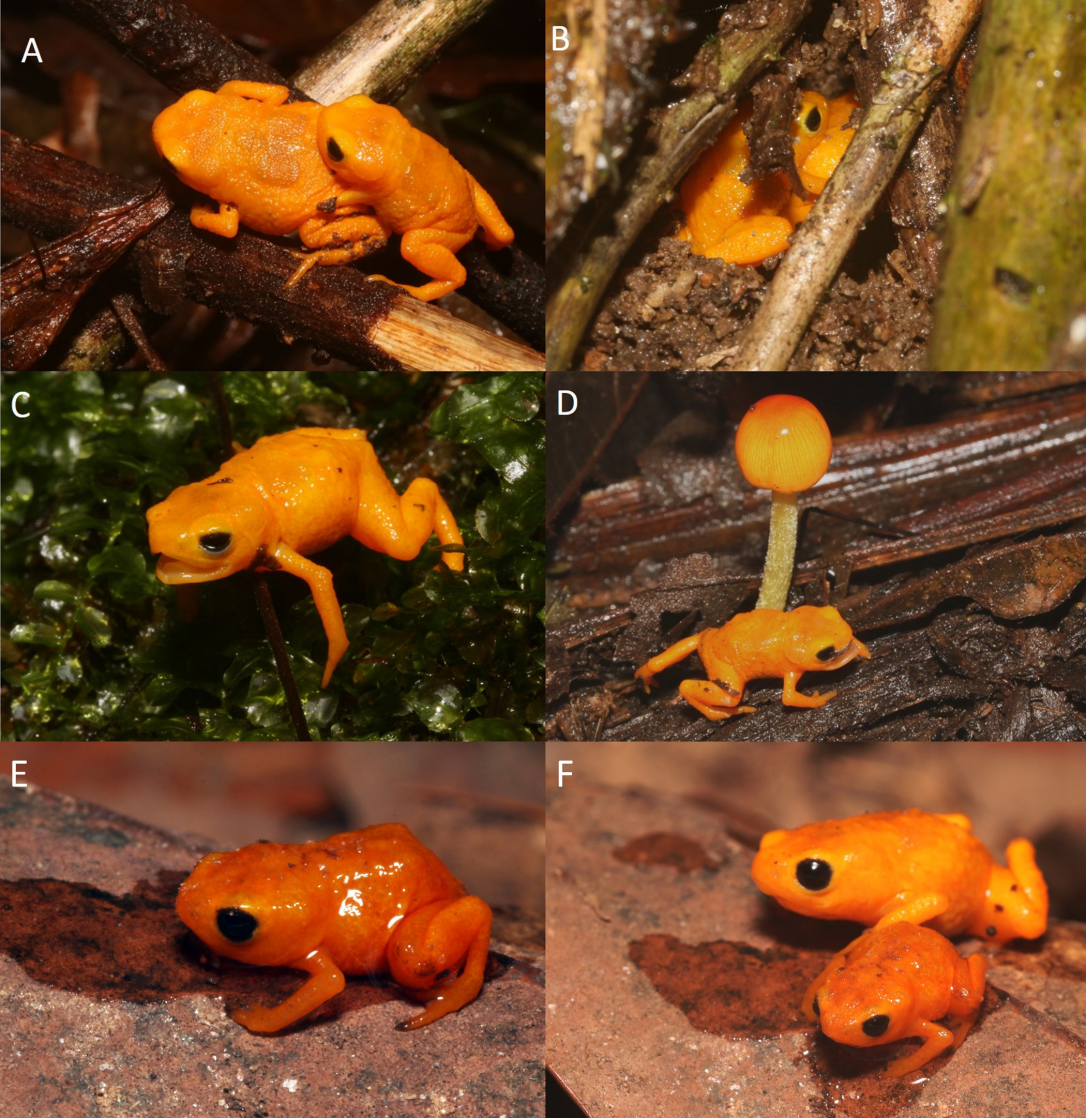
Natural history observations of *Brachycephalus rotenbergae* sp. nov. (A) Inguinal amplexus; (B) An amplected couple of hiding under the juçara palm (*E*. *edulis*) root system; (C) Mouth gaping defensive behaviour; (D) One specimen near a yellow/orange mushroom, notice the similarity of color and size; (E) One juvenile specimen; (F) Two juvenile specimens, but the smaller one is darker.

During this study, we found four amplexi, all of them the inguinal type (Fig 10A). In November 2018, we followed an amplected couple in the Projeto Dacnis area for five consecutive hours. During this time, the amplected couple climbed the slope terrain and stopped from time to time under some leaves. After a while, the couple entered under a juçara palm root system (Fig 10B). They went so deep into this shelter they were barely visible. After a few hours, four individuals emerged from the *E*. *edulis* root shelter: three distinctive bold females and one male. We searched for eggs at the site but did not find any. Considering that we observed this site during all the action, we strongly believe that the other two females were already inside the shelter before the couple arrived. Since we did not detect any clutch inside the shelter, we cannot say if the root system of juçara palm is used as a breeding site. Nonetheless, the presence of four different specimens under it is a strong evidence of its use at least as a shelter. We followed a second amplected couple on 5 February 2019, in Reserva das Araucárias area. The couple was moving through the landscape until it disappeared inside a huge root system. We waited to see if the couple would leave the site, without success. A third amplected couple was followed on the same day in the same area, but they separated after a few minutes of observation. The fourth amplexus occurred after two days of constant drizzling rain on 26 September 2019. Sadly, we unintentionally disturbed the couple, interrupting the amplexus. Despite the field work effort, we did not find any clutch deposition.

#### Defensive behaviour

We detected gaping defensive behaviour in three specimens that were manipulated, and we saw them opening their mouths widely (Fig 10C). The gaping and the flee away strategies were the two defensive displays that *Brachycephalus rotenbergae* **sp. nov.** presented during our study. The mouth-gaping behaviour was previously reported for *B*. *pitanga* and *B*. *ephippium* by Goutte et al. [68]. Despite the aposematic colours of *Brachycephalus rotenbergae* **sp. nov.**, it is important to note that their colours may also work as a camouflage in their microhabitat, since there are great amounts of tiny yellow and orange leaves, mushrooms and seeds on the ground (Fig 10D), especially during the active season. Together, these elements make the specimens’ bright colours not as conspicuous as they may seem. During our field surveys, it was quite common to mistake a specimen for other yellow/orange small things on the landscape.

In order to identify possible predators of *Brachycephalus rotenbergae* **sp. nov.**, we modelled tiny frog shapes using yellow and non-yellow atoxic modelling clay. We placed the frog models in some areas, recording any interaction with potential predators. During this assay, we recorded a slaty-breasted wood-rail (*Aramides saracura*) grasping a yellow frog model in its bill. The bird started running and dropped the model but picked it up from the ground. Although we cannot be sure if the bird recognized our model as a *Brachycephalus*, we suggest that yellow is not an efficient warning colour for *A*. *saracura*. During the study period, no predatory event on *Brachycephalus rotenbergae* **sp. nov.** was recorded.

#### Additional information

All study areas in São Francisco Xavier have problems with the invasive and exotic wild boar (*Sus scrofa*). Wild boar numbers are increasing in the subdistrict, and due to their foraging methods could negatively impact the *Brachycephalus rotenbergae* **sp. nov.** microhabitat, since these mammals excavate the soil and leaf litter in search of food. Despite no scientific evidence, we highlight the importance of future studies in evaluating the impact of this invasive species on frog communities.

## Discussion

The description of *Brachycephalus rotenbergae* **sp. nov.** is based on an integrative approach, coupling external morphology, coloration, osteology, vocalization, and genetic data. The *Brachycephalus* species has been taxonomically problematic owing to its morphological similarity and genetic conservatism [6, 22]. Currently, the literature has listed osteology, advertisement call and genetics as the main taxonomic informative data to describe new species for the genus *Brachycephalus* [23, 24]. The new species here described is genetically close to *B*. *margaritatus*. However, coloration with faded dark spots, absence of osteoderms, flat paravertebral plates, can easily diagnose *B*. *rotenbergae* **sp. nov.** from the latter. Furthermore, *Brachycephalus margaritatus* are restricted to the state of Rio de Janeiro [6, 64], as supported by our comparisons with congeners.

*Brachycephalus rotenbergae* **sp. nov.** has been referred to as *B*. *ephippium* for the state of São Paulo in the literature (e.g., [53, 69] for the type locality of the former), and scientific collection records. However, coloration with faded dark spots, presence of black connective tissue, parotic plate morphology, head wider than long, smaller size and 3% of genetic distance diagnose *B*. *rotenbergae* **sp. nov.** from *B*. *ephippium*. Also, *B*. *ephippium* is restricted to the state of Rio de Janeiro [6] with Mountains of Rio de Janeiro city suggested as type locality for the species; and we also have in the present study a robust number of specimens from several localities to support our comparisons with congeners.

Additionally, the duration of notes of the advertisement call of *Brachycephalus rotenbergae* **sp. nov.** seems to present slight differences for other localities of the State of São Paulo (cited as *Brachycephalus ephippium* [51]), with the “note B” showing higher values of pulses/s than the higher values of our recordings for this same acoustic parameter (100 pulses/s according to Fig 1B of [51]). The temporal parameters can be influenced by abiotic factors and the differences here seems to not be remarkable. Thus, further integrative investigation is needed to give proper description to the other vocalization records of *Brachycephalus rotenbergae* **sp. nov.** from the state of São Paulo. Moreover, the advertisement call of *Brachycephalus ephippium* (populations from the State of Rio de Janeiro), which would be valuable for further comparative studies, remains undescribed.

The genetic distance among the 16S rRNA mtDNA sequences of *B*. *ephippium* species group showed variation that helped us solve a puzzle. Considering both the 1% to 7% of variation for this fragment (present study) and the tree topology presented by Condez et al. [6], some conclusions are warranted. *Brachycephalus rotenbergae* **sp. nov.** is genetically divergent from all other species of the *B*. *ephippium* group, except *B*. *margaritatus*, with the phenotypic differences corroborating the specific status of the former. The low genetic divergence from *B*. *rotenbergae* **sp. nov.** to *B*. *margaritatus* can be due to (1) real genetic similarity, or (2) the 16S barcode is not effective to show the divergence between these populations. Several factors involved in the divergences at the population level can act in the magnitude of differences in intraspecific genetic divergences [70]. Thus, one gene fragment (here the 16S rRNA mtDNA fragment) for a recently derived species may not be very informative [71]. Recent splits have low support when studied by non-informative data (even for ultraconserved elements), as for *Brachycephalus* and *Melanophryniscus* [22]. For instance, Firkowski et al. [22] pointed out the differences in species delimitation according to the loci and methods and reinforced the need of several genes to have good resolutions for *Brachycephalus*. In addition, *B*. *rotenbergae* **sp. nov.** (called as *Brachyephalus* sp. 5, see [6]) and *B*. *margaritatus*, despite of the genetic distance, are reciprocally monophyletic.

The limits of the distribution of *Brachycephalus rotenbergae* **sp. nov.** overlaps the type locality of *B*. *atelopoide*. *Brachycephalus atelopoide* was described by Miranda-Ribeiro [55] based on only one specimen from municipality of Piquete, state of São Paulo, Brazil [4]. According to the original description (see [55], for the discussion of the information presented below), this would be a species without dorsal bone plates or bony projections on the spine, but with warts on the body. The holotype (by implication, not original designation) was collected among 31 specimens of *B*. *ephippium*. However, none of these remaining specimens agreed with the *B*. *atelopoide* description and were originally considered to be *B*. *ephippium*. Today, *B*. *atelopoide* cannot be associated with any of the populations associated with B. *ephippium* (by morphological similarity) or other *Brachycephalus* species (by distribution). Here, we have only one taxonomic information provided by the original description: the absence of dorsal bone plates. The first question that can be raised regards the ontogeny of dorsal bone plates. Miranda-Ribeiro [55] did not provide the size of the specimen. However, he also did not make any observations about the *B*. *atelopoide* specimen being smaller than the others or being a juvenile. In addition, the absence of these developed warts on the body in *B*. *rotenbergae* **sp. nov.** is a significant diagnosis from the original description of *B*. *atelopoide*. Thus, there is no information that would lead us to consider the lost holotype of *B*. *atelopoide* as a juvenile with absence of dorsal plates due to ontogeny. According to Pombal [13], among the specimens available, there are no individuals who agreed with the original description. The absence of information cannot be used here as a fact for the specimen developmental hypothesis. Therefore, the type cannot be traced, and we consider *B*. *atelopoide* a *species inquirenda*.

The fluorescence of the skull and post-cranial plates cannot be seen by the naked eye in juvenile specimens (less than SVL = 7.0 mm). The presence of different colour phases is not uncommon for anurans, including fluorescence (see [72, 73]). For instance, some species of the *Boana albopunctata* group have two colour phases, a remarkable ontogenetic change, with juveniles exhibiting a green background (potentially fluorescent), while adults have a brown background [73]. We suggest that absence of visible fluorescence in juveniles of *Brachycephalus rotengergae* **sp. nov.** is linked to the development of the co-ossified skull and post-cranial plates in adults. However, this statement is contingent on further analyses.

The natural history of *Brachycephalus rotenbergae* **sp. nov.** seems to show some conservative behaviours for the genus, as how specimens position themselves perched for vocalization (see [51, 73]), which brings some questions. *Brachycephalus* specimens are predominantly active by day on the forest floor and, occasionally, on low perches up to 50 cm [6, 51]. Males usually hold a territory for advertisements by acoustical and visual signals [51]. It has recently been discovered that some pumpkin toadlets are unable to listen to their own congeners and visual communication can be an important selective force for song being conserved in the lineages [68]. As these frogs have striking colours, they are diurnal and are probably well oriented visually, using higher perches helps intraspecific communication and consequently influences reproductive success in *Brachycephalus*. This would probably explain the use of higher perches as a vocalization site for species of the genus.

The inguinal amplectic position registered here for *B*. *rotenbergae* **sp. nov.** converges with data already presented in the literature (cited as *B*. *ephippium*; [51]), but we were not able to find oviposition sites. *Brachycephalus rotenbergae* **sp. nov.** and toads of the genus *Alytes* can shift to a more dorsal position during oviposition [51, 74]. This change to new position probably was the reason a cephalic amplexus was previously reported for *Brachycephalus* (see [75]), but not corroborated by the subsequent literature ([51, 76]; this study). The ancestral condition for the anuran amplexus seems to be inguinal, with the male clasping the female around the waist [74]. Considering most recent phylogenies for Anura [77], Brachycephalidae is the sister group of Eleutherodactylidae, and both are the sister clade of Craugastoridae. Thus, given that the axillary amplexus is widespread in the families Eleutherodactylidae and Craugastoridae [78, 79], we propose that *Brachycephalus* presents this form of amplexus by secondary derivation of this character, not by the retention of a plesiomorphic state. However, this statement needs further evolutionary analyses. The pumpkin toadlet can be locally abundant in many places, but natural history reports are scarce [51]. These reports are still scarce after 26 years, as previously observed by Wells [74], reinforcing that the natural history information presented herein is of notable importance for the knowledge of the group and future conservation efforts.

The geographic records for *B*. *rotenbergae* **sp. nov.** are here delimited to several localities at the south Mantiqueira mountain range and semidecidual forests in the municipalities of Mogi das Cruzes, Campinas and Jundiaí, with the Extent of Occurrence (EOO; [50]) being ~583.600 ha. It is common to the majority of the species of *Brachycephalus* to present a restricted distribution due to the geographic pattern for the genus [5, 80]. The sky islands scenario is supported by diversification-by-isolation and suggests that allopatric speciation due to climatic gradients is an important mechanism for generating species diversity and endemism in these regions [5, 7, 11, 22]. However, not all of the species of this genus presents such restricted distributions, like *B*. *nodoterga*. Despite the wider distribution for these two species, the geographic range is restricted to high places, like the other members of *Brachycephalus*, and it is expected that species with low dispersal capacity and poor plasticity regarding microhabitat and/or microclimate conditions are especially prone to diversify in these conditions [3, 5, 7]. Moreover, there are preserved areas near the type-locality and along the distribution polygon that could shelter the new species and possibly amplify its distribution.

Herein, we also presented several biological data. Despite the absence of population status, we have easily encountered this species in the type-locality and surrounding areas year by year. The habitat system is terrestrial, and the habitat type is forest. The district of São Francisco Xavier is mostly inserted in a Governmental Protected Area (Portuguese acronym APA), called São Francisco Xavier, and the Projeto Dacnis (and neighbours), has played an important role in hindering the continued decline in habitat area, extent and/or quality due to the residential and agricultural development. However, we call attention that the species was last found in the municipality of Campinas, São Paulo state, in February 1995 and is suggested to be locality extinct in that area (L.F. Toledo pers. comm.). Thus, we suggest the classification of *B*. *rotenbergae* **sp. nov.** as Least Concern (LC) category based on the IUCN Red List Categories and Criteria (Version 3.1; [81]), but highlight the sensitivity of these organisms to environmental disturbances. These data are significant to be used on the IUCN’s criteria and future lists of Brazilian threatened species.

## Supporting information

S1 File List of examined specimens. (DOCX)

S1 Table Genbank accession numbers. (DOC)

S2 Table Genetic distances between species of the *Brachycephalus ephippium* species group. (XLS)

S3 Table Record and respective sources for the geographic distribution map (Fig 5). (XLSX)

S1 Video Video recording of a male *Brachyecephalus rotenbergae* sp. nov. in calling activity. (MP4)
