# Supplementary material for: Hidden by the name: A new fluorescent pumpkin toadlet from the Brachycephalus ephippium group (Anura: Brachycephalidae)
Source: PLoS One. 2021 Apr 28;16(4):e0244812. doi: 10.1371/journal.pone.0244812 (PMC8081201; doi:10.1371/journal.pone.0244812)
Supplement: S1 File — (DOCX) [file pone.0244812.s001.docx]

**S1. Supporting Information 1.** Listo f additional examined specimens.

**APPENDIX.** Specimens examined.

*Brachycephalus alipioi*: Brazil, Espírito Santo, Castelo (MZUFV 5877–5882).

*Brachycephalus brunneus*: Brazil, Paraná, Campina Grande do Sul (MNRJ 40289–91, paratypes).

*Brachycephalus bufonoides*: Rio de Janeiro, Nova Friburgo (MZUSP 1459 lectotype).

*Brachycephalus darkside*: Brazil, Minas Gerais, Ervália (MZUFV 16636, holotype, MZUFV 15557–15561, 15565–15571, 15716–15721, 16491, 16579, 16627–16628,16631–16633, 16634–16635, 16780 paratypes, UFMG 19522 paratype, MNRJ 91327 paratype); Miradouro (MZUFV 2897, 6658–6660 paratypes).

*Brachycephalus didactylus*: Brazil, Rio de Janeiro, Paulo de Frontim (MNRJ 4067–73, paratypes).

*Brachycephalus ephippium*: Brazil, Rio de Janeiro, Rio de Janeiro (MNRJ 30919–29, 40782–807); Teresópolis (MNRJ 51580–83).

*Brachycephalus garbeanus*: Brazil, Rio de Janeiro, Nova Friburgo (MZUSP 0811 lectotype, MNRJ 17440–41, 25390–400, 67498).

*Brachycephalus hermogenesi*: Brazil, Paraná, Guaraqueçaba (MNRJ 87912).

*Brachycephalus izecksohni*: Brazil, Paraná, Quaratuba (MNRJ 76259–60, paratypes).

*Brachycephalus margaritatus*: Brazil, Rio de Janeiro, Petrópolis (MNRJ 85300–396).

*Brachycephalus nodoterga*: Brazil, São Paulo, São Paulo (MZUSP 0975, holotype).

*Brachycephalus pernix*: Brazil, Paraná, Quatro Barras (MNRJ 17343, holotype) and (MNRJ 17328–42, 17427–28, paratypes).

*Brachycephalus pitanga*: Brazil, São Paulo, São Luís do Paraitinga (MNRJ 60790–93, paratypes).

*Brachycephalus toby*: Brazil, São Paulo, Ubatuba (MNRJ 76382–83, paratypes).

*Brachycephalus tridactylus*: Brazil, Paraná, Guaraqueçaba (MNRJ 87908–910).

*Brachycephalus vertebralis*: Brazil, Rio de Janeiro, Paraty (MNRJ 11098, holotype) and (MNRJ 89199–201).
