# Supplementary material for: Hidden by the name: A new fluorescent pumpkin toadlet from the Brachycephalus ephippium group (Anura: Brachycephalidae)
Source: PLoS One. 2021 Apr 28;16(4):e0244812. doi: 10.1371/journal.pone.0244812 (PMC8081201; doi:10.1371/journal.pone.0244812)
Supplement: S1 Table — (DOC) [file pone.0244812.s002.doc]

**S1 Table.** Genbank accession numbers. Species IDs updated in the presente study.

| **GenBank acession number** | **Speciemen voucher** | **Species updated ID** | **Locality** | **State** | **Original Source** |
| --- | --- | --- | --- | --- | --- |
| MT396578 | HCLP-A79 | *Brachycephalus rotenbergae* sp. nov. | São Francisco Xavier | São Paulo | This study |
| MT396579 | HCLP-A78 | *Brachycephalus rotenbergae* sp. nov. | São Francisco Xavier | São Paulo | This study |
| MT396580 | HCLP-A80 | *Brachycephalus rotenbergae* sp. nov. | São Francisco Xavier | São Paulo | This study |
| KP999209 | Not given | *Brachycephalus rotenbergae* sp. nov. | São Francisco Xavier | São Paulo | Clemente-Carvalho et al., 2016 |
| KP999208 | Not given | *Brachycephalus rotenbergae* sp. nov. | São Francisco Xavier | São Paulo | Clemente-Carvalho et al., 2016 |
| KP999207 | Not given | *Brachycephalus rotenbergae* sp. nov. | São Francisco Xavier | São Paulo | Clemente-Carvalho et al., 2016 |
| KP999206 | Not given | *Brachycephalus rotenbergae* sp. nov. | São Francisco Xavier | São Paulo | Clemente-Carvalho et al., 2016 |
| KP999205 | Not given | *Brachycephalus rotenbergae* sp. nov. | São Francisco Xavier | São Paulo | Clemente-Carvalho et al., 2016 |
| KP999204 | Not given | *Brachycephalus rotenbergae* sp. nov. | São Francisco Xavier | São Paulo | Clemente-Carvalho et al., 2016 |
| KP999203 | Not given | *Brachycephalus rotenbergae* sp. nov. | São Francisco Xavier | São Paulo | Clemente-Carvalho et al., 2016 |
| HM216369 | CFBH16828 | *Brachycephalus rotenbergae* sp. nov. | São Francisco Xavier | São Paulo | Clemente-Carvalho et al., 2016 |
| KP999201 | Not given | *Brachycephalus rotenbergae* sp. nov. | Jundiaí | São Paulo | Clemente-Carvalho et al., 2016 |
| KP999200 | Not given | *Brachycephalus rotenbergae* sp. nov. | Jundiaí | São Paulo | Clemente-Carvalho et al., 2016 |
| KP999199 | Not given | *Brachycephalus rotenbergae* sp. nov. | Jundiaí | São Paulo | Clemente-Carvalho et al., 2016 |
| KP999198 | Not given | *Brachycephalus rotenbergae* sp. nov. | Jundiaí | São Paulo | Clemente-Carvalho et al., 2016 |
| KP999197 | Not given | *Brachycephalus rotenbergae* sp. nov. | Jundiaí | São Paulo | Clemente-Carvalho et al., 2016 |
| KP999196 | Not given | *Brachycephalus rotenbergae* sp. nov. | Jundiaí | São Paulo | Clemente-Carvalho et al., 2016 |
| KP999195 | Not given | *Brachycephalus rotenbergae* sp. nov. | Jundiaí | São Paulo | Clemente-Carvalho et al., 2016 |
| KP999194 | Not given | *Brachycephalus rotenbergae* sp. nov. | Jundiaí | São Paulo | Clemente-Carvalho et al., 2016 |
| KP999193 | Not given | *Brachycephalus rotenbergae* sp. nov. | Jundiaí | São Paulo | Clemente-Carvalho et al., 2016 |
| KP999192 | Not given | *Brachycephalus rotenbergae* sp. nov. | Atibaia | São Paulo | Clemente-Carvalho et al., 2016 |
| KP999191 | Not given | *Brachycephalus rotenbergae* sp. nov. | Atibaia | São Paulo | Clemente-Carvalho et al., 2016 |
| KP999190 | Not given | *Brachycephalus rotenbergae* sp. nov. | Atibaia | São Paulo | Clemente-Carvalho et al., 2016 |
| KP999189 | Not given | *Brachycephalus rotenbergae* sp. nov. | Atibaia | São Paulo | Clemente-Carvalho et al., 2016 |
| KP999188 | Not given | *Brachycephalus rotenbergae* sp. nov. | Atibaia | São Paulo | Clemente-Carvalho et al., 2016 |
| KP999187 | Not given | *Brachycephalus rotenbergae* sp. nov. | Atibaia | São Paulo | Clemente-Carvalho et al., 2016 |
| KP999186 | Not given | *Brachycephalus rotenbergae* sp. nov. | Atibaia | São Paulo | Clemente-Carvalho et al., 2016 |
| KP999185 | Not given | *Brachycephalus rotenbergae* sp. nov. | Atibaia | São Paulo | Clemente-Carvalho et al., 2016 |
| HQ435693 | CFBH16807 | *Brachycephalus rotenbergae* sp. nov. | Atibaia | São Paulo | Clemente-Carvalho et al., 2016 |
| HM216361 | CFBH16854 | *Brachycephalus rotenbergae* sp. nov. | Serra do japi | São Paulo | Clemente-Carvalho et al., 2016 |
| HM208306 | CFBH16809 | *Brachycephalus rotenbergae* sp. nov. | Atibaia | São Paulo | Clemente-Carvalho et al., 2016 |
| DQ283091 | CFBH2466 | *Brachycephalus rotenbergae* sp. nov. | Campinas | São Paulo | Clemente-Carvalho et al., 2016 |
| MK697367 | CFBH12355 | *Brachycephalus ephippium* | Parque Nacional da Serra dos Órgãos, Teresópolis | Rio de Janeiro | Condez et al., 2020 |
| MK697368 | MNRJ50260 | *Brachycephalus ephippium* | Vale da Revolta, Teresópolis | Rio de Janeiro | Condez et al., 2020 |
| MK697366 | MNRJ56517 | *Brachycephalus ephippium* | Reserva Ecologica de Guapiaçu | Rio de janeiro | Condez et al., 2020 |
| KJ649788 | IB6317 | *Brachycephalus nodoterga* | Not given | Not given | Clemente-Carvalho et al., 2016 |
| KJ649787 | IB6316 | *Brachycephalus nodoterga* | Not given | Not given | Clemente-Carvalho et al., 2016 |
| KJ649786 | IB6315 | *Brachycephalus nodoterga* | Not given | Not given | Clemente-Carvalho et al., 2016 |
| KJ649785 | IB6314 | *Brachycephalus nodoterga* | Not given | Not given | Clemente-Carvalho et al., 2016 |
| KJ649784 | IB6313 | *Brachycephalus nodoterga* | Not given | Not given | Clemente-Carvalho et al., 2016 |
| KJ649783 | IB6311 | *Brachycephalus nodoterga* | Not given | Not given | Clemente-Carvalho et al., 2016 |
| KJ649782 | IB6310 | *Brachycephalus nodoterga* | Not given | Not given | Clemente-Carvalho et al., 2016 |
| KJ649781 | IB634 | *Brachycephalus nodoterga* | Not given | Not given | Clemente-Carvalho et al., 2016 |
| KJ649780 | CA28413 | *Brachycephalus nodoterga* | Not given | Not given | Clemente-Carvalho et al., 2016 |
| KJ649779 | CA2226 | *Brachycephalus nodoterga* | Not given | Not given | Clemente-Carvalho et al., 2016 |
| KJ649778 | CA140 | *Brachycephalus nodoterga* | Not given | Not given | Clemente-Carvalho et al., 2016 |
| KJ649777 | BO138 | *Brachycephalus nodoterga* | Not given | Not given | Clemente-Carvalho et al., 2016 |
| KJ649776 | BO137 | *Brachycephalus nodoterga* | Not given | Not given | Clemente-Carvalho et al., 2016 |
| KJ649775 | BO136 | *Brachycephalus nodoterga* | Not given | Not given | Clemente-Carvalho et al., 2016 |
| KJ649774 | BO132 | *Brachycephalus nodoterga* | Not given | Not given | Clemente-Carvalho et al., 2016 |
| KJ649773 | BO131 | *Brachycephalus nodoterga* | Not given | Not given | Clemente-Carvalho et al., 2016 |
| KJ649772 | BO130 | *Brachycephalus nodoterga* | Not given | Not given | Clemente-Carvalho et al., 2016 |
| KJ649771 | BO128 | *Brachycephalus nodoterga* | Not given | Not given | Clemente-Carvalho et al., 2016 |
| KJ649771 | CFBH6211 | *Brachycephalus nodoterga* | Serra da Cantareira | São Paulo | Clemente-Carvalho et al., 2011 |
| MK697375 | MNRJ76100 | *Brachycephalus margaritatus* | Paty de Alferes | Rio de Janeiro | Condez et al., 2020 |
| MK697376 | MNRJ73701 | *Brachycephalus margaritatus* | Petrópolis | Rio de Janeiro | Condez et al., 2020 |
| MK697377 | CFBH2420 | *Brachycephalus margaritatus* | Petrópolis | Rio de Janeiro | Condez et al., 2020 |
| HQ435690 | CFBH3566 | *Brachycephalus alipioi* | Vargem alta | Espirito Santo | Clemente-Carvalho et al., 2011 |
| HQ435699 | CFBH16746 | *Brachycephalus pitanga* | São Luís do Paraitinga | São Paulo | Clemente-Carvalho et al., 2011 |
| HQ435701 | CFBH23002 | *Brachycephalus toby* | Ubatuba | São Paulo | Clemente-Carvalho et al., 2011 |
| HQ435694 | CFBH16800 | *Brachycephalus garbeanus* | Macaé de cima | Rio de Janeiro | Clemente-Carvalho et al., 2011 |
| HM216365 | CFBH16801 | *Brachycephalus garbeanus* | Macaé de cima | Rio de Janeiro | Clemente-Carvalho et al., 2016 |
| MK697361 | CFBH27859 | *Brachycephalus crispus* | Cunha | São Paulo | Condez et al., 2020 |
| MK697362 | CFBH44400 | *Brachycephalus crispus* | Cunha | São Paulo | Condez et al., 2020 |
| MK697371 | CFBH12997 | *Brachycephalus guarani* | Prumirim, Ubatuba | São Paulo | Condez et al., 2020 |
| HQ435702 | CFBH7907 | *Brachycephalus vertebralis* | Ubatuba | São Paulo | Clemente-Carvalho et al., 2011 |
| MH259788 | ZUFRJ15428 | *Brachycephalus bufunoides* | Lumiar, Nova Friburgo | Rio de Janeiro | Folly et al., 2020 |
| MH259789 | ZUFRJ15425 | *Brachycephalus bufunoides* | Lumiar, Nova Friburgo | Rio de Janeiro | Folly et al., 2020 |
| MH259790 | ZUFRJ15424 | *Brachycephalus bufunoides* | Lumiar, Nova Friburgo | Rio de Janeiro | Folly et al., 2020 |
| MH259791 | ZUFRJ15426 | *Brachycephalus bufunoides* | Lumiar, Nova Friburgo | Rio de Janeiro | Folly et al., 2020 |
| MH259792 | ZUFRJ15429 | *Brachycephalus bufunoides* | Lumiar, Nova Friburgo | Rio de Janeiro | Folly et al., 2020 |
